# Corticotropin releasing factor (CRF) systems: Promoting cocaine pursuit without distress via incentive motivation

**DOI:** 10.1371/journal.pone.0267345

**Published:** 2022-05-03

**Authors:** Hannah M. Baumgartner, Madeliene Granillo, Jay Schulkin, Kent C. Berridge

**Affiliations:** 1 Department of Psychology, University of Michigan Ann Arbor, Ann Arbor, Michigan, United Started of America; 2 School of Medicine, University of Washington, Seattle, Washington, United States of America; University of Kentucky, UNITED STATES

## Abstract

Corticotropin releasing factor (CRF) systems in limbic structures are posited to mediate stress-induced relapse in addiction, traditionally by generating distress states that spur drug consumption as attempts at hedonic self-medication. Yet evidence suggests that activating CRF-expressing neurons in the central amygdala (CeA) or nucleus accumbens (NAc) can magnify incentive motivation in absence of distress, at least for sucrose rewards. However, traditional CRF hypotheses in addiction neuroscience are primarily directed toward drug rewards. The question remains open whether CRF systems can similarly act via incentive motivation mechanisms to promote pursuit of drug rewards, such as cocaine. Here we tested whether optogenetic excitation of CRF-containing neurons in either NAc medial shell, lateral CeA, or dorsolateral BNST of transgenic *Crh*-Cre+ rats would spur preference and pursuit of a particular laser-paired cocaine reward over an alternative cocaine reward, and whether excitation served as a positively-valenced incentive itself, through laser self-stimulation tests. We report that excitation of CRF-containing neurons in either NAc or CeA recruited mesocorticolimbic circuitry to amplify incentive motivation to pursue the laser-paired cocaine: focusing preference on the laser-paired cocaine reward in a two-choice task, and spurred pursuit as doubled breakpoint in a progressive ratio task. Crucially indicating positive-valence, excitation of CRF neurons in NAc and CeA also was actively sought after by most rats in self-stimulation tasks. Conversely, CRF neuronal activation in BNST was never self-stimulated, but failed to enhance cocaine consumption. Collectively, we find that NAc and CeA CRF-containing neurons can amplify pursuit and consumption of cocaine by positively-valenced incentive mechanisms, without any aversive distress.

## Introduction

Stress can increase drug consumption and exacerbate relapse in addiction [1–3]. Diverse stressors activate neurons that release corticotropin releasing factor (CRF). Traditional addiction neuroscience theories have posited that activation of CRF-containing neurons generates negatively-valenced distress states that drive drug consumption and addictive relapse, as attempts to counteract the negative state [4–11].

For example, the hyperkatifeia/allostasis neuroscience theory of addiction, derived from psychological opponent-process theory, posits that activation of CRF-containing neurons in central amygdala (CeA) and bed nucleus of stria terminalis (BNST) produces negative distress, including feelings of drug withdrawal as well as general life distress [6–8, 12, 13].

However, despite ample evidence that CRF systems can contribute to negative distress, other findings suggest that CRF-containing neurons in CeA and nucleus accumbens (NAc) alternatively have positively-valenced or incentive-mediated psychological routes to cause intense pursuit and consumption of rewards [14–18]. For example, optogenetic stimulation of CRF-containing neurons in CeA and NAc of C*rh*-Cre+ rats recruited reward-related mesocorticolimbic circuitry to increase incentive motivation for sucrose reward, and narrowly-focused pursuit on laser-paired sucrose [19]. Demonstrating positive-valence, most of those rats actively worked for laser to self-stimulate their CRF-containing neurons in CeA and NAc.

Such observations indicate that CRF neural systems in CeA and NAc may increase incentive motivation for sucrose reward via a positively-valenced process [19]. Incentive salience or ‘wanting’ is a specific mesolimbic-mediated motivational process that attributes positive incentive value to neural representations of rewards and their cues. Incentive salience attribution makes such reward-related stimuli become attractive, and able to elicit preference, pursuit and consumption. The incentive-sensitization theory of addiction posits that drugs that induce mesolimbic sensitization of accumbens dopamine-related systems cause excessive ‘wanting’ to take drugs in susceptible individuals without increasing drug ‘liking’, even in the absence of distress [20, 21]. Thus, excessive incentive salience provides, at least in principle, an alternative positively-valenced motivational route by which CRF neurons in CeA or NAc could recruit mesolimbic circuitry to cause addictive pursuit, drug consumption and relapse. That was the primary question to be explored here.

By contrast to CeA and NAc, in the BNST (an output target of CeA), similar optogenetic excitations of CRF-containing neurons caused an apparently aversive state that rats avoided [19], partially consistent with the allostatic hypothesis that CRF activation in BNST mediates distress. Yet, the aversive state generated by activation of BNST CRF neurons suppressed motivation for sucrose rewards rather than increased motivation to consume rewards.

However, hyperkatifeia/allostasis and related opponent-process hypotheses of CRF function in addiction were originally intended to explain motivation for drug rewards, not sucrose rewards [6–8, 12, 13]. It remains unknown whether the CRF neuronal incentive role found for sucrose [19] would transfer to motivation for a drug of abuse, such as cocaine. We therefore tested here whether optogenetic excitation of CRF-containing neurons in NAc, CeA or BNST would similarly act via positively-valenced incentive motivation processes to increase pursuit and consumption of intravenous cocaine rewards. We paired optogenetic excitation of CRF-containing neurons in either NAc, CeA, or BNST of BAC transgenic *Crh*-Cre rats with a particular opportunity to earn intrajugular infusions of cocaine in a two-choice task: one cocaine option paired with laser stimulation vs an equal cocaine option occurring alone, similar to a previous study [22]. In a progressive ratio task, we further asked if CRF neuronal stimulation in NAc, CeA or BNST increased the intensity of incentive motivation, expressed as effort breakpoint, to self-administer cocaine. Finally, we assessed the motivational valence of exciting CRF-containing neurons alone by determining whether *Crh*-Cre rats would self-administer laser stimulation in either NAc, CeA or BNST. Together with cross-brain Fos measures of neural recruitment, our results indicate that CRF-containing neural systems in NAc and CeA recruit mesocorticolimbic circuitry to amplify and focus incentive motivation to pursue and consume cocaine.

## Materials and methods

### Animals

Female (n = 23) and male (n = 19) transgenic *Crh*-Cre+ rats were bred and genotyped in house, from breeders obtained from the Messing laboratory at the University of Texas [23]. Rats were housed in same-sex pairs at 21C° under reverse light cycle (lights-off 8am; testing began 1–3 hours after lights-off) with ad libitum access to food and water until catheter implantation surgery (>3 months old). Rats were then single-housed and maintained on a restricted food schedule (85–90% previous body weight) for the duration of cocaine self-administration experiments. All experimental procedures were approved by the University of Michigan Institutional Animal Care & Use Committee in accordance with NIH animal care and use guidelines.

### Optogenetic surgery

Rats were anesthetized with isoflurane gas for surgery (induction: 4–5%, maintenance, 1–2%) and placed in a stereotaxic apparatus (David Kopf Instruments, Tujunga, CA). Atropine (0.05 mg/kg; i.p.; Henry Schein) was given at start of surgery and cefazolin (75 mg/kg, s.c.; Henry Schein) and carprofen (5 mg/kg; s.c.; Henry Schein) were given immediately after surgery. Additional carprofen injections were repeated post-surgically for two-days during recovery.

Bilateral microinjections (1μl per side) into either NAc medial shell, lateral CeA, or dorsolateral BNST were made of either active AAV-DIO-ChR2-eYFP virus (n = 23; UNC Vector Core) with ChR2 or inactive control virus AAV-DIO-eYFP (n = 19). Co-expression of *Crh* and *Cre* mRNAs in NAc, CeA, and BNST neurons was previously validated using fluorescent *in situ* hybridization for this transgenic line [19]. Both the active ChR2 virus and the inactive eYFP-only virus were driven by an EF1a promoter to infect only neurons that express Cre-recombinase.

Stereotaxic site coordinates for virus microinjections were bilaterally identical for individual rats but were staggered across rats in the following ranges so that each group’s sites filled most of NAc medial shell, lateral CeA, and dorsolateral BNST: NAc medial shell range (from bregma): A/P: +1.08 to +2.52, M/L: ±0.6 to 1.8, D/V: -6.0 to -7.2 (Angle used: 16 or 10 degrees, flat skull; n = 13); lateral CeA range: A/P: -1.92 to -3.24, M/L: ± 3.6 to 4.6, D/V: -6.8 to -8.4 (flat skull; n = 13); dorsolateral BNST range, A/P: -0.36 to +0.36, M/L: 1.4 to 1.8, D/V: -6.0 to -6.5, (Angle used: 16 degrees, flat skull; n = 17; S1 Table in S1 File). A 1.0 μl volume of virus per hemisphere was microinjected at each bilateral site over a 10-min period (0.1 μl / min), and the microinjector was left in place for an additional 10-min to allow diffusion. Optic fibers (200 μm) were bilaterally implanted in the same surgery and placed so that each fiber tip was aimed 0.3mm dorsal to virus microinjection site and secured with skull screws and dental cement.

### Intrajugular catheter surgery

Approximately 2 weeks after stereotaxic surgeries, rats again received pre- and post-operative treatments as described above, and underwent an additional surgery to insert an intravenous catheter into the jugular vein for cocaine self-administration experiments [22]. Briefly, a subcutaneous anchor was secured in the mid-scapular region and its attached Silastic catheter was passed subcutaneously up the back and dorsal neck region, and threaded into the right jugular vein [22]. Following surgeries, rats received daily intra-catheter gentamicin infusions for 10 days to prevent infection, as well as daily intra-catheter infusions of heparinized saline that continued throughout testing. Rats were given 7–10 days for recovery from surgery prior to behavioral testing. Catheter patency was confirmed with brevital sodium injections (0.2ml, 20mg/ml) before behavioral testing and again after completion of all self-administration tests were completed (i.e., twice total per rat). Rats that failed to become ataxic within 10s were excluded from self-administration analyses.

### Laser parameters

Optogenetic blue laser (473nm) excitation was delivered at 10Hz (10ms-ON-90ms-OFF; 2-3mW) [19, 24], using durations described below.

### Two-choice cocaine preference task

An instrumental two-choice task tested whether pairing laser excitation of CRF-containing neurons in NAc, CeA, or BNST with earning an i.v. cocaine reward made that infusion more or less valuable than an identical cocaine infusion delivered without laser [22]. Briefly, rats were trained to nose poke into two retractable portholes on a fixed-ratio 1 schedule for i.v. cocaine self-administration. A nose poke into one assigned porthole (*Laser+Cocaine*) would deliver 8-sec of laser stimulation together with an intrajugular infusion of cocaine (0.3mg/kg for each rat, 50ul volume dissolved in a sterile saline solution, 2.8-sec per infusion, National Institute on Drug Abuse) and 8-sec of a distinct paired sound cue (tone or white noise). A nose poke into the *Cocaine-alone* porthole earned an identical cocaine infusion and alternate paired sound cue, but no laser. Both portholes retracted for a 20-sec timeout following each infusion earned from either porthole.

Test sessions started with several single-choice trials, in which only one porthole was presented at a time (*Laser+Cocaine* or *Cocaine-alone)* and then was retracted after a nosepoke. A single-choice trial with the other porthole was then offered. Both single-choice trials were then repeated once more. Following these 4 single-choice exposures, two-choice trials were offered for the remainder of the 1-hr daily session, allowing rats to choose either porthole. Rats that failed to earn at least 5 daily cocaine infusions on days 1–3 were excluded from analyses (4 of 36).

### Progressive ratio test

To test whether laser excitation of CRF-containing neurons changed the *magnitude* of incentive motivation for cocaine rewards, rats underwent two days of progressive ratio (PR) testing in 1-hr sessions. On one day, only the *Laser+Cocaine* porthole was available, and on the other day only the *Cocaine-alone* porthole was available (order counter-balanced). Earning an infusion increased the number of responses required to earn the next cocaine infusion on a PR schedule (progressive ratio schedule = 1, 2, 4, 6, 9, 12, 15, 20, 25, 32, 40, 50, 62, 77, 95, 118, 145, 178, 219, 268, etc.; PR = [5*e*^(*reward number* × 0.2)^] − 5) [22]. The effort breakpoint reached when responding stopped was compared between conditions (i.e., the maximum effort price paid in terms of responses to earn cocaine within each 1-hr session).

### Spout-touch laser self-stimulation

To evaluate the incentive value of laser by itself, rats could earn laser illuminations (3-sec) by touching an empty waterspout designated as *Laser-spout*. Touching an alternative *Inactive-spout* delivered nothing, as a control measure of exploration. Self-stimulation sessions (30min) occurred over 3 days. On day 1 rats were assessed for self-stimulation criteria and days 2–3 were statistically analyzed for consistency. *Robust self-stimulation* criterion required earning ≥50 laser illuminations and at least twice as many touches on *Laser-spout* as on *Inactive-spout*. *Low self-stimulation* criterion required at least ≥10 laser illuminations, with twice as many touches on *Laser-spout* as on *Inactive-spout* [19, 25].

### Histology

Histology and immunohistochemistry followed previously reported procedures [19]. Rats underwent a final 30-min laser stimulation session for Fos inducement that ended 45 minutes before transcardial perfusions. Brains were extracted, sectioned into 40μm slices (Leica, Wetzlar, Germany), mounted onto slides, and stained for Fos protein and GFP expression. Briefly, tissue was rinsed three times for 10min in sodium phosphate buffer (NaPB) and blocked in 5% normal donkey serum (60min) before overnight incubation in rabbit anti-cFos (1:2500; Catalog#: 226 003; Lot #: 4–63; RRID:AB_2231974; Synaptic Systems, Göttingen, Germany) and chicken anti-GFP (1:2000; Catalog#: AB13970; Lot #: GR3190550-30; RRID:AB_300798; Abcam, Cambridge, MA). Slices were again rinsed 3x in NaPB for 10min and placed for 2 hours in biotinylated donkey anti-rabbit (1:300; Catalog #: AB2340593; Lot #: 128703; RRID: AB2340593; Jackson Immunoresearch, West Grove, PA) and donkey anti-chicken AlexaFluor 488 (1:300; Code #: AB2340375; Lot #: 144438; RRID:AB_2340375; Jackson Immunoresearch, West Grove, PA). Following 3 more rinses in NaPb, tissue was then incubated for 90min in tertiary containing Streptavidin Cy3 (1:300; Catalog #: AB2337244, Lot #: 141873, RRID: AB_2337244; Jackson Immunoresearch, West Grove, PA), before three final 10min rinses. Slices were mounted onto slides (Fischer), coverslipped with Prolong-gold with DAPI (Invitrogen), and imaged using a digital camera (Qimaging, Surrey, BC, Canada) attached to a fluorescent microscope (Leica, Wetzlar, Germany). Viral expression of ChR2 virus was confirmed and visualized using filter cubes with excitation bands of 490-510nm.

### Assessment of distant Fos recruitment

Coronal whole-brain images (10x) were used to manually count Fos+ neurons using previous methods [19, 26]. Functional connectivity recruited by CRF ChR2 stimulation, reflected as changes of neural activity in distant brain structures, was measured by Fos protein expression in mesocorticolimbic structures: anteromedial orbitofrontal cortex (OFC), infralimbic cortex (IF), anterior NAc shell (aNAcSh), posterior NAcSh (pNAcSh), NAc core (NAcC), anterior ventral pallidum (aVP), PVP, anterior BNST (aBNST), pBNST, anterior lateral hypothalamus (aLH), pLH, paraventricular nucleus of the hypothalamus (PVN), basolateral amygdala (BLA), CeA, medial amygdala (MeA), ventral tegmentum (VTA), substantia nigra (SN), and midbrain periaqueductal grey (PAG). In each structure targeted in coronal whole-brain images (10x magnification) 3 boxes for counting Fos+ neurons ranging in size between 6–49 μm^2^ were placed at approximately the same subregional location in each rat in the structure, for CRF ChR2 rats, control eYFP rats, and control naïve unoperated rats [27]. Raw cell counts of Fos+ neurons in each structure were compared between three groups in a one-way ANOVA followed by Bonferonni corrected t-tests. Box size was tailored for each structure to contain ~10 Fos+ neurons in naïve unoperated brain tissue. Percent enhancement in Fos expression recruited in each structure by NAc, CeA, or BNST laser illumination in *Crh*-Cre+ ChR2 rats was calculated by comparison to equivalent structures in inactive eYFP control rats that received similar laser illuminations prior to euthanasia.

### Statistical analysis

Behavioral data were analyzed using mixed-model ANOVAs with between-subject (i.e., ChR2/eYFP) and/or within-subject factors (i.e., days), followed by paired t-tests with Bonferroni corrections (IBM, SPSS Statistics). Wilcoxon Z was used for nonparametric data. Independent pairwise comparisons were used for distant Fos analysis. For all analyses p = 0.05, two-tailed. Cohen’s D and $r=\frac{\text{Z}}{\sqrt{\text{N}1+\text{N}2}}$ were used to calculate effect sizes.

## Results

### Local Fos plumes around illuminated CRF ChR2 fibers

**Fig 1:**
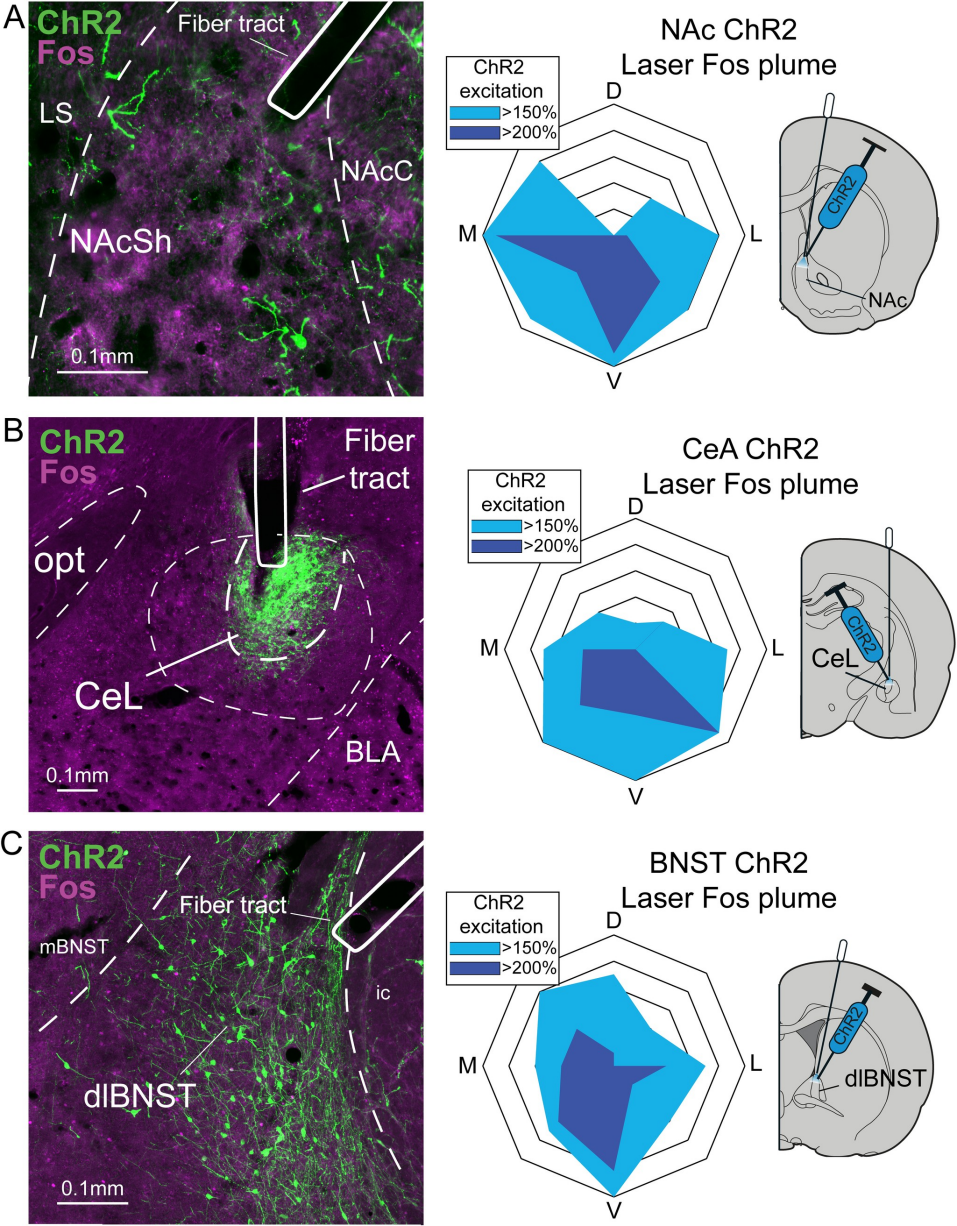
Photomicrograph of virus expression and local Fos plumes. Photomicrographs display ChR2 virus expression (green) in neurons of *Crh*-Cre rats, and laser-induced local Fos expression in neurons (purple) located within plumes surrounding optogenetic fiber tips (mapped at right). Fiber/virus sites are in A) nucleus accumbens (NAc) medial shell, B) central nucleus of the amygdala (CeA), and C) bed nucleus of the stria terminalis (BNST). We previously reported co-expression of *Crh* mRNA and *Cre* mRNAs in the same neurons within NAc, CeA and BNST using fluorescent *in situ* hybridization to validate this transgenic *Crh-*Cre rat line (19). Laser Fos plume diagrams at right show the average plume diameter and % elevation intensity of local Fos expression immediately surrounding the fiber tips, induced by laser stimulation of CRF-containing neurons in CRF ChR2 rats, compared to control levels measured at the same box sites in eYFP rats after identical laser illuminations. Light blue reflects >150% Fos elevation and dark blue reflects >200% Fos elevation over eYFP baseline levels measured in the inactive virus control group. Scale bars show 0.1mm for reference. NAcSh, nucleus accumbens shell; NAcC, nucleus accumbens core; LS, lateral septum; opt, optic tract; CeL, lateral central amygdala; BLA, basolateral amygdala; mBNST, medial bed nucleus of stria terminalis; dlBNST, dorsolateral bed nucleus of stria terminalis.

**Fig 2:**
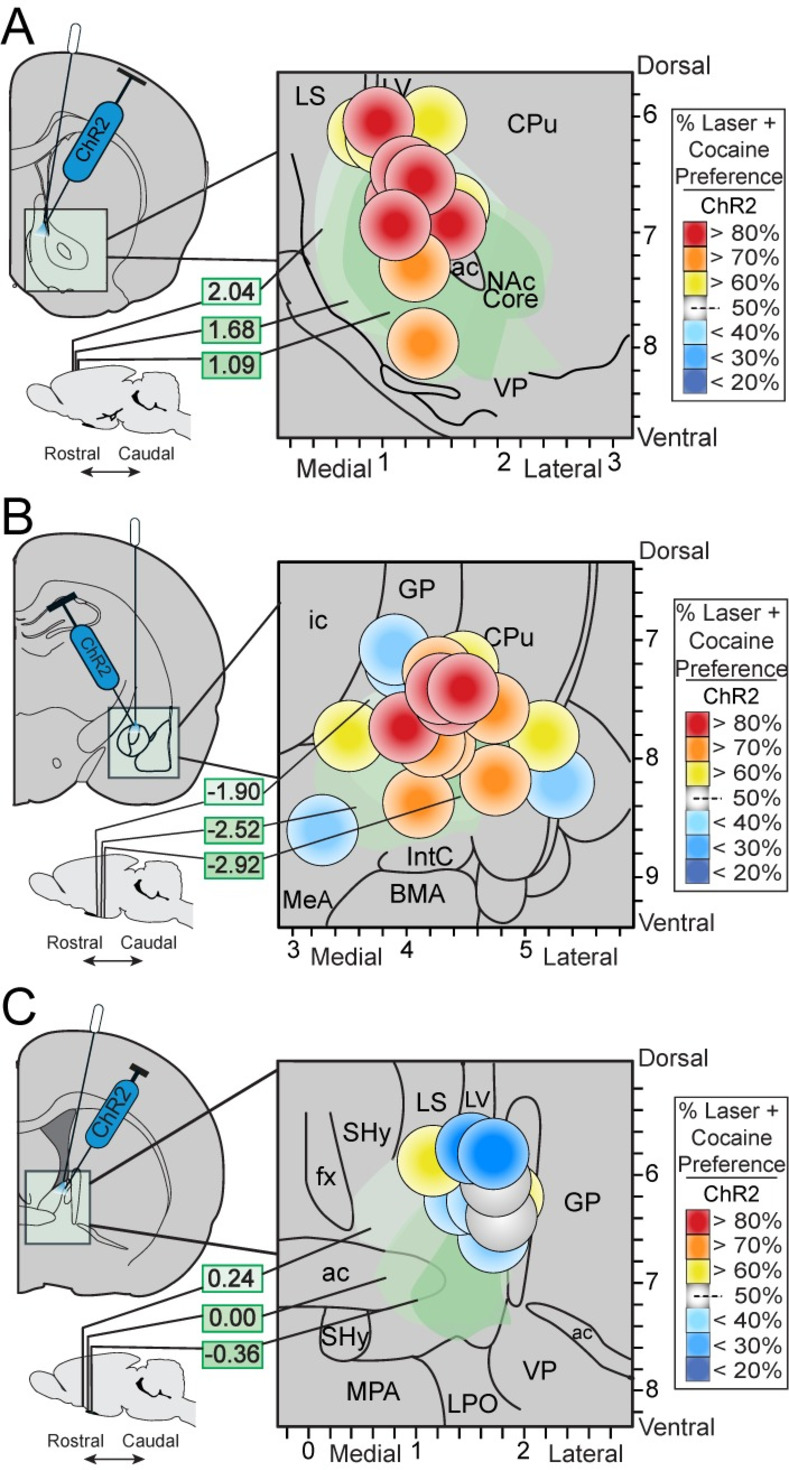
Localization of function maps for *Laser+Cocaine* preference. Localization of function maps show the magnitude of behavioral effects induced at corresponding histological sites of optic fibers in individual ChR2 CRF rats: **A**) nucleus accumbens (NAc) medial shell, **B**) central nucleus of the amygdala (CeA), and **C**) dorsolateral bed nucleus of the stria terminalis (BNST). Colors indicate intensity (%) of laser-induced pursuit with reds indicating stronger preference for *Laser+Cocaine* in the two-choice task. Conversely, blue colors indicate avoidance of *Laser+Cocaine* (or preference for *Cocaine alone*). Symbol sizes are scaled to match maximum 0.7mm diameter measured for illuminated ChR2 Fos plumes. Also see S1 Table in S1 File. LS, lateral septum; LV, lateral ventricle; CPu, caudate putamen; NAc, nucleus accumbens; VP, ventral pallidum; ac, anterior commissure; ic, internal capsule; MeA, medial amygdala; GP, globus pallidus; IntC, intercalated amygdala; BMA, basomedial amygdala; BLA, basolateral amygdala; fx, fornix; Shy, septohypothalamic nucleus, MPA, medial preoptic area; LPO, lateral preoptic area.

At sites in NAc, CeA, and BNST, ChR2 laser stimulation of CRF-expressing neurons in CRF ChR2 rats produced local Fos plumes of 0.17–0.36mm radius immediately surrounding illuminated optic fiber tips, containing 150–200% elevation of Fos expression above eYFP control levels at equivalent sites. The size of these radii suggests that laser illumination locally activated ChR2-infected CRF-containing neurons in zones of approximately ~0.4–0.7 mm diameter similarly in all three structures (Fig 1). Therefore the 0.7mm maximal diameter size was used to depict local neuronal activations for placement symbols in localization-of-function maps (Fig 2).

### Distant Fos activation in mesocorticolimbic circuitry

**Fig 3:**
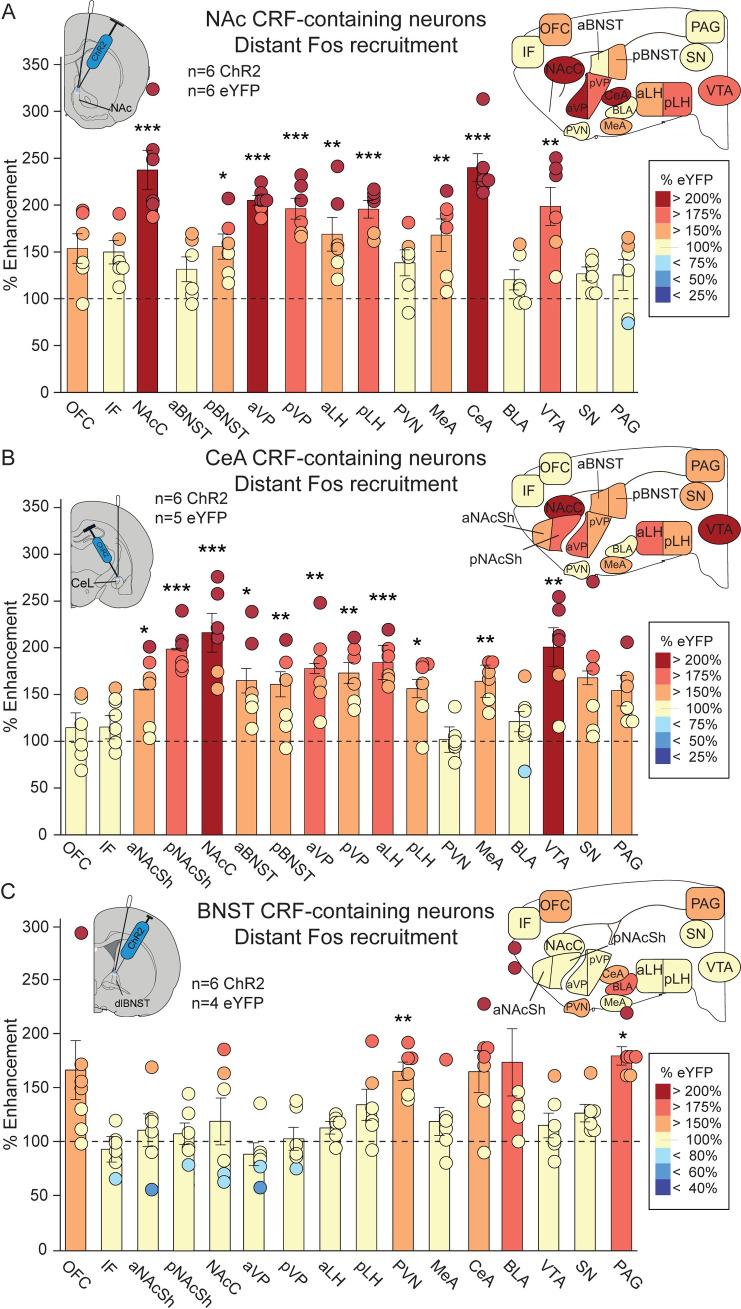
Distant Fos recruitment of mesocorticolimbic activity. **A**) After NAc medial shell ChR2 excitation of CRF-containing neurons, distant 150–300% increases in Fos expression over eYFP control levels were recruited in several mesocorticolimbic structures related to incentive motivation for rewards: nucleus accumbens core (NAcC), anterior and posterior ventral pallidum (aVP, pVP), posterior bed nucleus of stria terminalis (pBNST), anterior and posterior lateral hypothalamus (aLH, pLH), medial amygdala (MeA), central amygdala (CeA), and ventral tegmentum (VTA). **B**) After CeA ChR2 excitation of CRF neurons, 150–300% increases in distant Fos were recruited in: NAcC, anterior and posterior NAc shell (aNAcSh, pNAcSh), aVP, pVP, anterior BNST (aBNST), pBNST, aLH, pLH, MeA and VTA. **C)** In contrast for BNST, ChR2 excitation of CRF-containing neurons recruited 150–300% increases in Fos in several distinct structures related to avoidance/pain/distress: paraventricular nucleus of the hypothalamus (PVN) and midbrain periaqueductal grey (PAG). All analyses were performed on raw cell counts (see S2-S4 Tables in S1 File). Bar graphs depict means and SEMs of percent change from eYFP control rats and colors denote the degree of change. Circles show individual ChR2 CRF rats. SN, substantia nigra; IF, infralimbic cortex. *p<0.05, **p<0.01, ***p<0.001.

In NAc ChR2 rats (n = 6; Fig 3A and Table 1 and S2 Table in S1 File), laser stimulation of CRF-expressing neurons recruited >150–350% increases in distant Fos expression in several mesocorticolimbic structures: NAcC, aVP, pVP, aLH, pLH, pBNST, MeA, CeA, and VTA.

**Table 1:** Brain-wide Fos activation following CRF-containing neuron excitation in NAc.

NAc CRF neurons	Fos+ count Mean ± SEM			ANOVA F-statistic, p-value		NAc ChR2 vs. eYFP Bonferroni adjusted p-value		
Region	Naïve control (n = 4)	NAc ChR2 (n = 6)	NAc eYFP (n = 6)	*F*	*p*	*p*	95% CI	*d*
IF	22.3 ± 2.6	22.5 ± 2.1	15 ± 2.1	3.729	*0*.*052*			
OFC	58 ± 5.2	55.3 ± 4.2	42.2 ± 4.2	3.586	*0*.*058*			
NAcC	32 ± 5.9	69.7 ± 4.8	29.3 ± 4.8	20.772	*<0*.001***	*<0*.001***	22, 59	3.12
aVP	16 ± 2.5	53 ± 2.1	2.8 ± 2.1	76.282	*<0*.001***	*<0*.001***	19, 35	5.08
pVP	30.3± 3.7	50.7 ± 3.1	25.8 ± 3.1	18.619	*<0*.001***	*<0*.001***	13, 37	3.17
aBNST	31.5 ± 11	40.3 ± 9.9	30.7 ± 4.9	2.196	*0*.*151*			
pBNST	29.5 ± 6.4	58.7 ± 5.2	37.7 ± 5.2	7.318	*0*.007**	*0*.04*	1, 41	1.49
aLH	19.5 ± 4.2	46.2 ± 3.4	27.3 ± 3.4	13.748	*0*.001**	*0*.006**	5, 32	2.26
pLH	25 ± 3.6	46 ± 2.9	23.5 ± 2.9	17.78	*<0*.001***	*<0*.001***	11, 34	3.21
PVN	21 ± 3.7	37.3 ± 2.9	27 ± 2.9	6.664	*0*.01**	*0*.*083*		
MeA	23.5 ± 4.6	51.5 ± 3.8	30.7 ± 3.8	12.965	*0*.001**	*0*.006**	6, 36	2.32
CeA	13.8 ± 2.4	36 ± 2	15 ± 2	34.77	*<0*.001***	*<0*.001***	13, 29	4.20
BLA	22 ± 3.7	32.7 ± 3	27.2 ± 3	2.495	*0*.*121*			
VTA	20 ± 4.9	53.2 ± 4	26.8 ± 4.9	16.33	*0*.001**	*0*.005**	9, 44	2.76
SN	16 ± 1.7	21.5 ± 1.4	17 ± 1.7	3.669	*0*.*06*			
PAG	26 ± 4.4	28.8 ± 3.6	23 ± 5	0.451	*0*.*649*			

Table shows Fos+ protein quantification in mesocorticolimbic regions after final exposure to ChR2 excitation in NAc (top; n = 3 female, n = 3 male). Fos+ protein quantification in mesocorticolimbic regions (left columns) for naïve unoperated control rats, ChR2 rats, and eYFP rats, were compared with a one-way ANOVA for each structure targeted. “Fos+ Count” reflects mean of each group at each site ± standard error (SEM). Follow up two-tailed t-tests with Bonferonni corrections were performed between ChR2, eYFP, and naiive control groups. The right column includes *p-*values, confidence intervals, and effect sizes for instances where ChR2 rats were found to significantly differ from eYFP controls. Percent change in ChR2 rats from eYFP controls are depicted in Fig 3. IF, infralimbic cortex; OFC, orbitofrontal cortex; aNAcSh, anterior nucleus accumbens shell; pNAcSh, posterior nucleus accumbens shell; NAcC, nucleus accumbens core; aVP, anterior ventral pallidum; pVP, posterior ventral pallidum; aBNST, anterior bed nucleus of stria terminalis; pBNST, posterior bed nucleus of stria terminalis; aLH, anterior lateral hypothalamus; pLH, posterior lateral hypothalamus; PVN, hypothalamic paraventricular nucleus; MeA, medial amygdala; CeA, central amygdala; BLA, basolateral amygdala; VTA, ventral tegmentum; SN, substantia nigra; PAG, midbrain periaqueductal gray.

*p<0.05,

**p<0.01,

***p<0.001

In CeA ChR2 rats (n = 6), laser excitation of CRF-containing neurons similarly recruited >150–350% increases in distant Fos expression in several of the same structures: NAcC, aNAcSh, pNAcSh, aVP, pVP, aLH, aBNST, pBNST, MeA, and VTA (Fig 3B and Table 2, and S3 Table in S1 File).

**Table 2:** Brain-wide Fos activation following CRF-containing neuron excitation in CeA.

CeA CRF neurons	Fos+ count Mean ± SEM			ANOVA F-statistic, p-value		CeA ChR2 vs. eYFP Bonferroni adjusted p-value		
Region	Naïve control (n = 4)	CeA ChR2 (n = 6)	CeA eYFP (n = 5)	*F*	*p*	*p*	95% CI	*d*
IF	22.3 ± 3	22.8 ± 2.4	19.8 ± 2.7	0.377	*0*.*693*			
OFC	58 ± 7.1	53.2 ± 5.8	46.4 ± 6.3	0.77	*0*.*485*			
NAcC	32 ± 7.2	83 ± 5.9	38.4 ± 6.4	19.8	*<0*.001***	*<0*.001***	20, 68	2.80
aNAcSh	38.5 ± 4.6	55.7 ± 3.7	35.8 ± 4.1	7.6	*0*.007**	*0*.011*	4, 35	2.44
pNAcSh	39 ± 4.5	87 ± 3.6	43.8 ± 4	47.011	*<0*.001***	*<0*.001***	28, 58	4.34
aVP	16 ± 4.9	50.2 ± 4	28.2 ± 4.4	15.684	*<0*.001***	*0*.009**	6, 39	1.98
pVP	30.3± 3.7	55.7 ± 3.1	32.2 ± 3.3	19.139	*<0*.001***	*0*.001**	11, 36	3.33
aBNST	31.5 ± 4.9	43.5 ± 4	26.4 ± 4.4	4.459	*0*.036*	*0*.041*	1, 34	2.46
pBNST	29.5 ± 5	60.2 ± 4.1	37.4 ± 4.5	13.133	*0*.001**	*0*.008**	6, 40	2.09
aLH	19.5 ± 2.7	45.3 ± 2.2	24.6 ± 2.4	34.472	*<0*.001***	*<0*.001***	12, 30	3.91
pLH	25 ± 4.1	45.3 ± 3.3	29 ± 3.6	9.154	*0*.004**	*0*.019*	3, 30	2.04
PVN	21 ± 3.8	35.7 ± 3.1	35 ± 3.4	5.203	*0*.024*	*>0*.*05*		
MeA	23.5 ± 3.2	45.3 ± 2.7	27.6 ± 2.9	16.783	*<0*.001***	*0*.002**	7, 29	2.81
BLA	22 ± 5.8	39.2 ± 4.7	32.4 ± 5.1	2.646	*0*.*112*			
VTA	20 ± 3.9	43.3 ± 3.2	21.6 ± 3.5	14.621	*0*.001**	*0*.002**	8, 35	2.76
SN	16 ± 3.6	25.5 ± 2.9	15.2 ± 3.2	3.413	*0*.*067*			
PAG	26 ± 4.4	33 ± 3.2	21.4 ± 3.5	2.977	*0*.*089*			

Table shows Fos+ protein quantification in mesocorticolimbic regions after final exposure to ChR2 excitation in CeA (n = 3 female, n = 3 male). Fos+ protein quantification in mesocorticolimbic regions (left columns) for naïve unoperated control rats, ChR2 rats, and eYFP rats, were compared with a one-way ANOVA for each structure targeted. “Fos+ Count” reflects the mean of each group at each site ± standard error (SEM). Follow up two-tailed t-tests with Bonferonni corrections were performed between ChR2, eYFP, and naiive control groups. The right column includes *p-*values, confidence intervals, and effect sizes for instances where ChR2 rats were found to significantly differ from eYFP controls. Percent change in ChR2 rats from eYFP controls are depicted in Fig 3. IF, infralimbic cortex; OFC, orbitofrontal cortex; aNAcSh, anterior nucleus accumbens shell; pNAcSh, posterior nucleus accumbens shell; NAcC, nucleus accumbens core; aVP, anterior ventral pallidum; pVP, posterior ventral pallidum; aBNST, anterior bed nucleus of stria terminalis; pBNST, posterior bed nucleus of stria terminalis; aLH, anterior lateral hypothalamus; pLH, posterior lateral hypothalamus; PVN, hypothalamic paraventricular nucleus; MeA, medial amygdala; CeA, central amygdala; BLA, basolateral amygdala; VTA, ventral tegmentum; SN, substantia nigra; PAG, midbrain periaqueductal gray.

*p<0.05,

**p<0.01,

***p<0.001

By contrast in BNST ChR2 rats, laser excitation CRF-containing neurons (n = 6) failed to recruit comparably intense Fos increases in reward-related NAc, VP, VTA or LH structures. Instead BNST CRF neuronal stimulation recruited >150–200% increases in Fos in other structures linked to distress or pain, such as PVN and PAG, as well as producing marginal trends toward increase in CeA, BLA and OFC (Fig 3C and Table 3 and S4 Table in S1 File).

**Table 3:** Brain-wide Fos activation following CRF-containing neuron excitation BNST.

BNST CRF neurons	Fos+ count Mean ± SEM			ANOVA F-statistic, p-value		BNST ChR2 vs. eYFP Bonferroni adjusted p-value		
Region	Naïve control (n = 4)	BNST ChR2 (n = 6)	BNST eYFP (n = 4)	*F*	*p*	*P*	95% CI	*d*
IF	22.3 ± 3.6	17.1 ± 3	18.3 ± 3.6	0.619	*0*.*556*			
OFC	58 ± 8.1	52.3 ± 6.7	33.8 ± 8.1	2.503	*0*.*127*			
NAcC	32 ± 8.9	48.8 ± 7.3	40.1 ± 8.9	1.077	*0*.*374*			
aNAcSh	38.5 ± 6.1	35.5 ± 4.9	31.8 ± 6.1	0.313	*0*.*738*			
pNAcSh	39 ± 5.7	47.7 ± 4.7	44 ± 5.7	0.695	*0*.*52*			
aVP	16 ± 3	27.5 ± 2.4	30.8 ± 3	6.919	*0*.011*	*>0*.*05*		
pVP	30.3± 3.7	38.2 ± 3.1	36.8 ± 3.7	1.425	*0*.*282*			
aLH	19.5 ± 2.8	29.7 ± 2.3	28 ± 2.8	4.129	*0*.046*	*>0*.*05*		
pLH	25 ± 3.8	34.8 ± 3.2	25.8 ± 3.9	2.583	*0*.*120*			
PVN	21 ± 2.3	34.5 ± 1.9	20.8 ± 2.3	14.453	*0*.001**	*0*.003**	5, 22	3.61
MeA	23.5 ± 4.2	33.8 ± 3.4	28.3 ± 4.2	1.877	*0*.*199*			
CeA	13.8 ± 2.4	20.3 ± 1.9	12.3 ± 2.4	4.142	*0*.046*	*0*.*071*		
BLA	22 ± 5.9	28 ± 4.8	21.8 ± 5.9	3.212	*0*.*08*			
VTA	20 ± 4.7	35.2 ± 3.8	30.3 ± 4.7	3.206	*0*.*08*			
SN	16 ± 2	21.7 ± 1.7	17 ± 2	2.806	*0*.*104*			
PAG	26 ± 3.2	31.2 ± 2.7	17.3 ± 3.3	5.381	*0*.023*	*0*.022*	1, 26	3.55

Table shows Fos+ protein quantification in mesocorticolimbic regions after final exposure to ChR2 excitation in BNST (n = 3 female, n = 3 male ChR2 group). Fos+ protein quantification in mesocorticolimbic regions (left columns) for naïve unoperated control rats, ChR2 rats, and eYFP rats, were compared with a one-way ANOVA for each structure targeted. “Fos+ Count” reflects mean of each group at each site ± standard error (SEM). Follow up two-tailed t-tests with Bonferonni corrections were performed between ChR2, eYFP, and naiive control groups. The right column includes *p-*values, confidence intervals, and effect sizes for instances where ChR2 rats were found to significantly differ from eYFP controls. Percent change in ChR2 rats from eYFP controls are depicted in Fig 3. IF, infralimbic cortex; OFC, orbitofrontal cortex; aNAcSh, anterior nucleus accumbens shell; pNAcSh, posterior nucleus accumbens shell; NAcC, nucleus accumbens core; aVP, anterior ventral pallidum; pVP, posterior ventral pallidum; aBNST, anterior bed nucleus of stria terminalis; pBNST, posterior bed nucleus of stria terminalis; aLH, anterior lateral hypothalamus; pLH, posterior lateral hypothalamus; PVN, hypothalamic paraventricular nucleus; MeA, medial amygdala; CeA, central amygdala; BLA, basolateral amygdala; VTA, ventral tegmentum; SN, substantia nigra; PAG, midbrain periaqueductal gray.

*p<0.05,

**p<0.01,

***p<0.001

### NAc and CeA CRF neurons focus ‘wanting’ for laser-paired cocaine in two-choice task

#### NAc CRF neuronal stimulation in two-choice task

**Fig 4:**
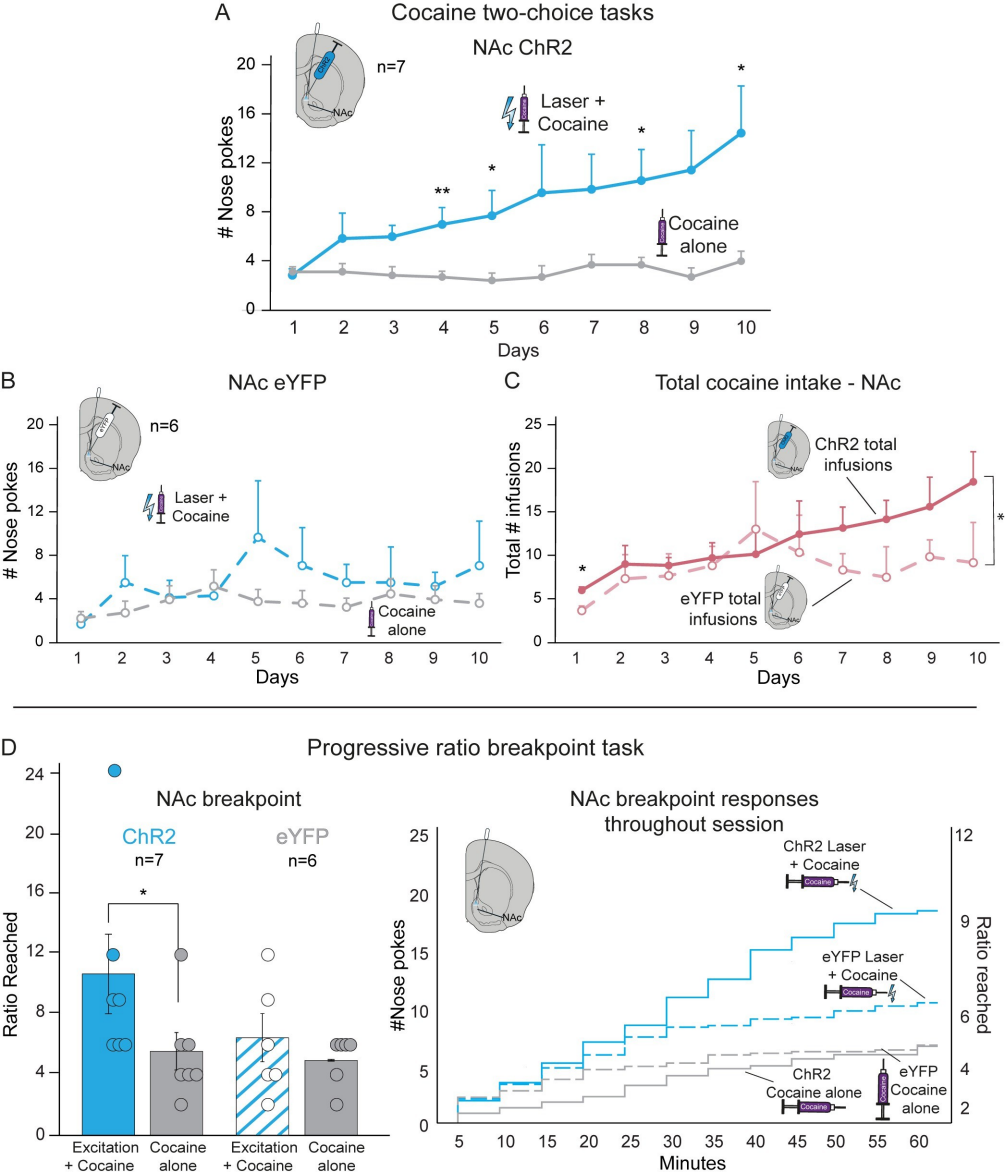
NAc ChR2 stimulation of CRF-containing neurons amplifies motivation and focuses preference on laser-paired cocaine. **A)** In the two-choice task, ChR2 excitation of CRF-containing neurons in NAc medial shell focused preference and cocaine pursuit on the *Laser+Cocaine* option by a 4:1 ratio over *Cocaine alone* in the two-choice task (n = 7). **B)** In comparison, control NAc eYFP rats chose equally between the two cocaine options (n = 6). **C)** NAc ChR2 rats also escalated total cocaine intake (*Laser+Cocaine* plus *Cocaine alone* options combined) to higher magnitudes than eYFP controls in two-choice task. **D)** In the progressive ratio (PR) task that measures effort breakpoint, NAc CRF-containing neuron excitation nearly doubled breakpoint in the *Laser+Cocaine* session over the *Cocaine alone* session (n = 7; left panel). Laser did not affect the breakpoint of NAc eYFP control rats (n = 6). Right panel depicts timeframe of behavioral responses for cocaine during the 1hr PR sessions. Means and SEM reported. *p<0.05, **p<0.01.

For ChR2 rats (n = 7) with virus/fiber sites in NAc, ChR2 laser stimulation paired with one cocaine option in the two-choice task caused rats to pursue that *Laser+Cocaine* option by a 4:1 ratio over their relatively ignored *Cocaine-alone* option across test days (*F*_1,6_ = 6.970, *p* = 0.039; Fig 4A). Consequently, NAc ChR2 rats earned 13.8±3.0 cocaine infusions on average from the *Laser+Cocaine* porthole on day 10 versus only 3.3±0.6 *Cocaine-alone* infusions (*t*_6_ = 3.035, *p* = 0.023, *d* = 3.16, 95% CI:[1.9,17.2]). Unlike NAc ChR2 rats, control NAc eYFP rats with inactive virus (n = 6) chose essentially equally between *Laser+Cocaine* and *Cocaine-alone* options across all test days (*F*_1,5_ = 1.367, *p* = 0.295; Fig 4B). NAc ChR2 rats also escalated their overall cocaine intake across the 10 test days, reaching magnitudes nearly twice as high as final intake levels of NAc eYFP control rats (*F*_9,3_ = 9.555, *p* = 0.045; Fig 4C).

#### CeA CRF neuronal stimulation in two-choice task

**Fig 5:**
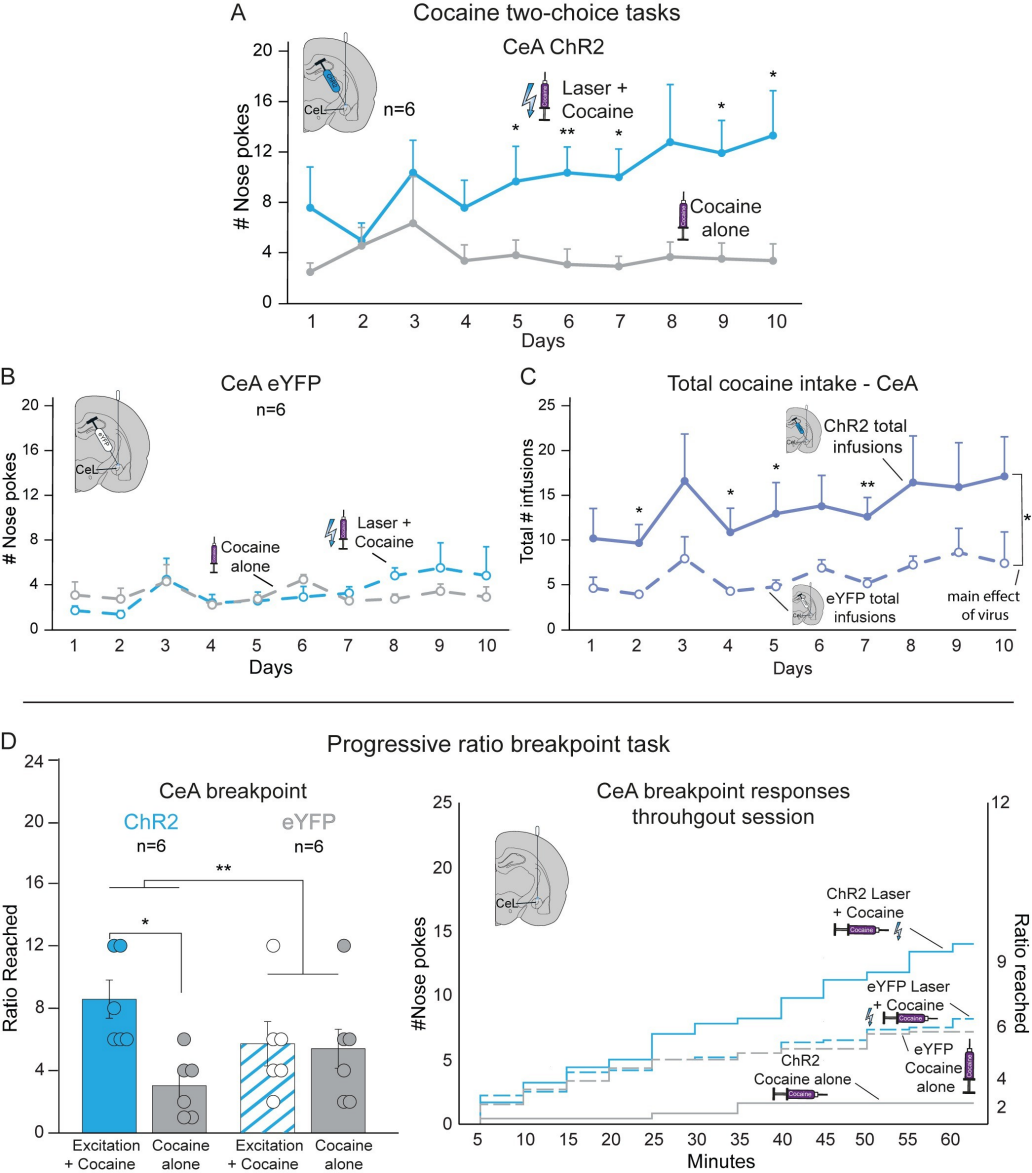
CeA ChR2 stimulation of CRF-containing neurons amplifies motivation and focuses preference on laser-paired cocaine. **A)** In the two-choice task, ChR2 excitation of CRF-containing neurons in the lateral CeA amplified cocaine pursuit and focused preference on the *Laser+Cocaine* option by a 4:1 ratio over *Cocaine alone* test (n = 6). **B)** In comparison, control CeA eYFP rats chose equally between options (n = 6). **C)** CeA ChR2 rats also had higher total cocaine intake in the two-choice task across all days than eYFP controls (combined intakes from both *Laser+Cocaine* plus *Cocaine alone*). **D)** In a separate progressive ratio (PR) breakpoint test of incentive motivation intensity, CeA CRF neuronal excitation doubled effort breakpoint for cocaine in the *Laser+Cocaine* session over breakpoint of the same CeA ChR2 rats in their *Cocaine alone* session (n = 6; left panel). Laser did not affect breakpoint of CeA eYFP control rats (n = 6). Right panel depicts timeframe of behavioral responses for cocaine during the 1hr PR sessions. Means and SEM reported. *p<0.05, **p<0.01.

For CeA ChR2 rats, excitation of CRF-containing neurons in the two-choice task (n = 6) focused cocaine pursuit on the *Laser+Cocaine* option by a 4:1 ratio over the *Cocaine-alone* option across testing (*F*_1,5_ = 14.669, *p* = 0.012; Fig 5A). On day 10 CeA ChR2 rats earned 12.8±3.4 *Laser+Cocaine* infusions versus only 3.8±1.3 *Cocaine-alone* infusions (*t*_5_ = 3.022, *p* = 0.029, *d* = 1.58, 95% CI:[1.3,16.7]). By contrast, CeA eYFP control rats chose equally between cocaine options across all test days (*F*_1,5_ = 0.540, *p* = 0.495; Fig 5B). CeA ChR2 stimulation also appeared to escalate total cocaine consumption to higher magnitudes than CeA eYFP controls throughout testing (*F*_1,10_ = 10.329, *p* = 0.009; Fig 5C).

### NAc and CeA CRF neurons amplify cocaine breakpoint motivation

A PR task measured the magnitude of incentive motivation, expressed as maximum breakpoint that rats were willing to exert for cocaine as price increased throughout each session.

Laser excitation of CRF-containing neurons in NAc doubled effort breakpoint to obtain cocaine in NAc ChR2 rats, which reached 10.4±2.6 nosepokes per infusion for on the *Laser+Cocaine* day versus breakpoints of only 5.4±1.2 on the *Cocaine-alone* day (n = 7; Wilcoxon Z = 2.207, *p* = 0.027, *r* = 0.83; Fig 4D). NAc ChR2 rats made 22.4±8.5 total nosepokes on the *Laser+Cocaine* day, or 300% more than on the *Cocaine-alone* day (7.9±2.9; Z = 2.336, *p* = 0.018, *r* = 0.89). In contrast, NAc eYFP control rats (n = 6) did not differ in their breakpoints between test days (Z = 0.677, *p* = 0.498).

For CeA ChR2 rats, excitation of CRF-containing neurons doubled their effort to obtain cocaine, which reached breakpoints of 8.0±1.0 nosepokes per infusion on the *Laser+Cocaine* day, or >200% higher than on the *Cocaine-alone* day (3.0±0.8; n = 6; Z = 2.207, *p* = 0.027, *r* = 0.90; Fig 5D). CeA ChR2 rats also made 15.2±3.6 nosepokes on the *Laser+Cocaine* day versus 3.2±1.5 nosepokes on the *Cocaine-alone* day (Z = 2.226, *p* = 0.026, *r* = 0.91). In contrast, CeA eYFP control rats (n = 6) did not differ in breakpoints between the PR test days (Z = 0.962, *p* = 0.336), and therefore significantly differed from CeA ChR2 rats in breakpoint pattern (*F*_1,10_ = 16.900, *p* = 0.002).

### BNST CRF stimulation fails to alter cocaine preference

**Fig 6:**
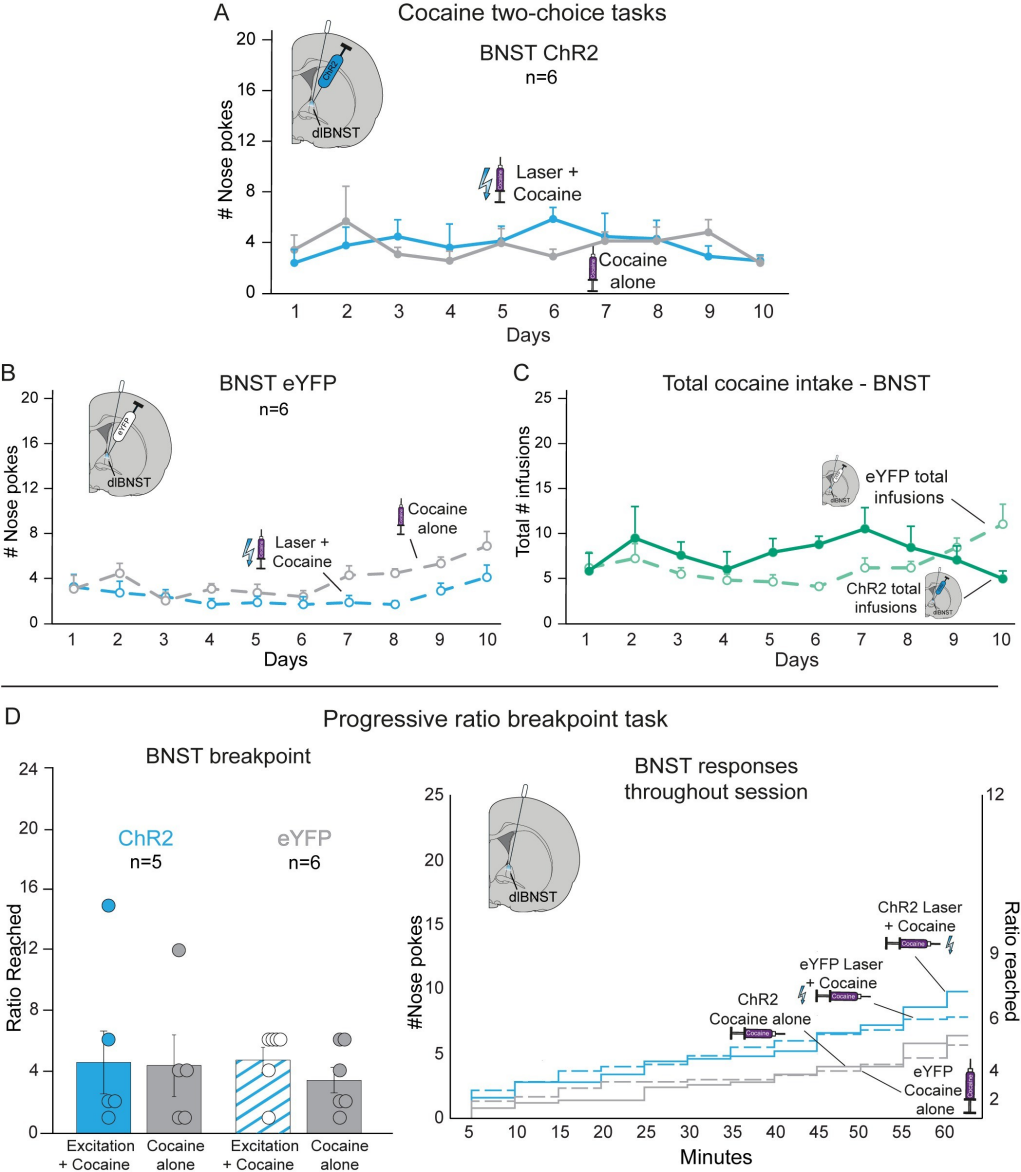
BNST ChR2 stimulation of CRF-containing neurons fails to change cocaine pursuit. Pairing ChR2 excitation of CRF neurons in BNST with one cocaine option failed to alter cocaine pursuit in the two-choice task for **A)** BNST ChR2 rats (n = 6); or **B)** BNST eYFP inactive control rats, (n = 6). **C)** BNST ChR2 stimulation had little no effect on total cocaine consumption in the two-choice task compared to eYFP controls plus *Cocaine alone* infusions). **D)** In progressive ratio (PR) tests, CRF-containing neuronal excitation failed to alter effort breakpoint of BNST ChR2 rats (n = 5; left panel), which remained similar also to the unchanging breakpoint of BNST eYFP control rats (n = 6). Right panel depicts timeframe of behavioral responses for cocaine during the 1hr PR sessions. Means and SEM reported.

For BNST ChR2 rats in the two-choice task, paired laser excitation of CRF-containing neurons in BNST (n = 6) did not alter preference between the *Laser+Cocaine* and *Cocaine-alone* options. BNST ChR2 rats chose equally between cocaine options at nearly a 1:1 ratio across all test days (*F*_1,5_ = 0.000, *p* = 1.000; Fig 6A). Similarly, BNST eYFP control rats pursued *Laser+Cocaine* and *Cocaine-alone* options equally throughout testing (*F*_1,5_ = 3.773, *p* = 0.110; Fig 6B and 6C).

Across the 10 test days, BNST ChR2 rats consumed only one-third of the total cocaine on average than the amount consumed by either NAc or CeA ChR2 rats in the two-choice task (*F*_1,17_ = 7.496, *p* = 0.014).

#### BNST CRF stimulation does not alter breakpoint

In the progressive ratio task, laser excitation of CRF neurons in BNST failed to alter breakpoint effort for cocaine, as BNST ChR2 rats reached similar effort breakpoints on the *Laser+Cocaine* day (5.2±2.6) and on the *Cocaine-alone* day (4.3±2.0; n = 5 Wilcoxon Z = 0.921, *p* = 0.357; Fig 6D). Control BNST eYFP rats also reached similar breakpoints for *Laser+Cocaine* and *Cocaine-alone* (n = 6; Z = 0.962, *p* = 0.336).

### Laser self-stimulation: NAc and CeA CRF groups

In the spout-touch laser self-stimulation task, rats could earn 3-sec bins of laser illumination by touching a designated empty *Laser-spout*, while touching the other *Inactive-spout* earned nothing as a control measure of exploration. Rats were classified as *robust self-stimulators* if they made ≥50 *Laser-spout* touches and twice as many *Laser-spout* as *Inactive-spout* touches. Rats were classified as *low self-stimulators* if they made at least ≥10 (but <50) touches on *Laser-spout* and twice as many *Laser-spout* as *Inactive-spout* touches. Rats that met neither criterion were classified as failing to self-stimulate. Self-stimulation status was initially classified on day 1 and was retested for consistency on days 2–3.

#### NAc: Low CRF laser self-stimulation

**Fig 7:**
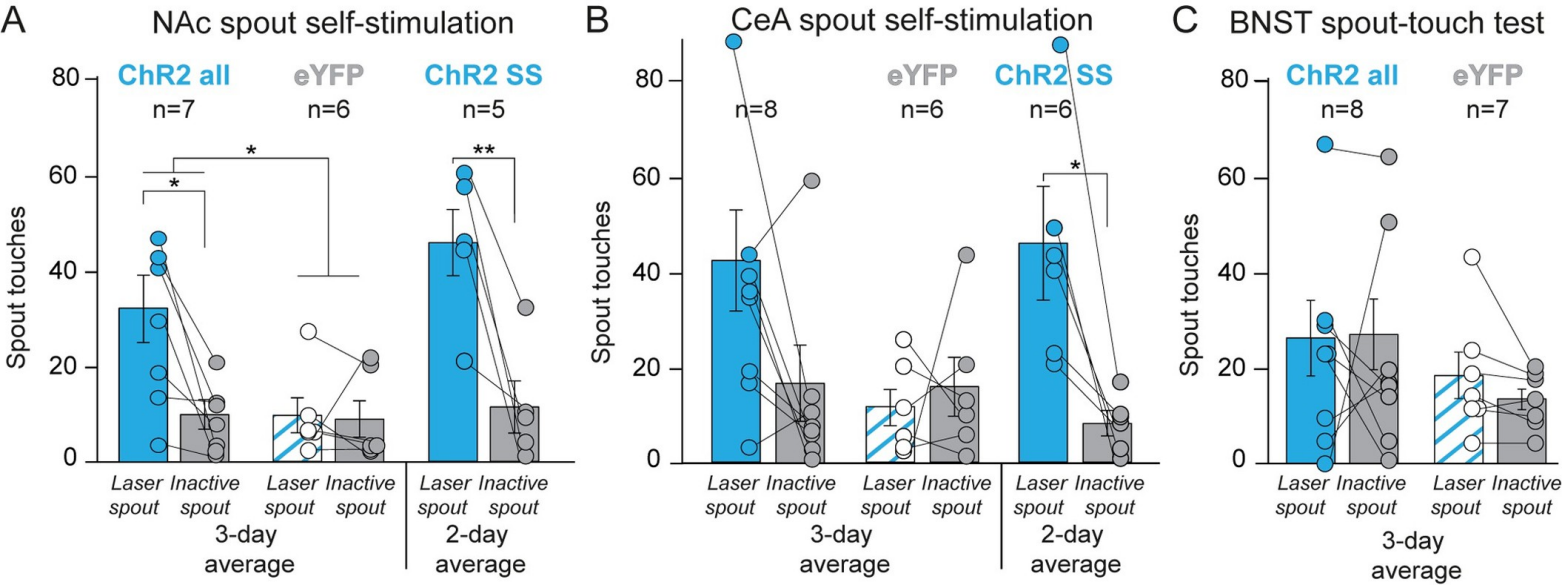
Laser self-stimulation of CRF-containing neurons in NAc and CeA, but not BNST. **A)** ChR2 rats could self-stimulate laser to excite CRF-containing neurons by touching a designated *Laser spout* to earn 3sec illuminations, whereas touching an *Inactive spout* delivered nothing. NAc ChR2 rats as an entire group demonstrated significant self-stimulation across all 3 days of testing (n = 7). NAc ChR2 individuals that met criteria for laser self-stimulation on day 1 (n = 5, ChR2 SS) continued to self-stimulate on days 2–3 by touching their *Laser-spout >*400% more than the *Inactive-spout*. **B)** CeA ChR2 rats as an entire group showed only a nonsignificant trend toward laser self-stimulation (n = 8). However, CeA ChR2 individuals that met self-stimulation criteria on day 1 (n = 6; ChR2 SS) continued to self-stimulate on days 2–3. touching their *Laser-spout* 400% more than the *Inactive-spout*. By contrast, control NAc eYFP (n = 6) and CeA eYFP rats (n = 6) failed to self-stimulate, and merely touched randomly at low rates. **C)** BNST ChR2 rats failed as a group to self-stimulate for laser excitation of CRF-containing neurons (n = 8) across all 3 test days, similar to eYFP controls (n = 7). No BNST ChR2 individuals met criteria for self-stimulation on any day. Means and SEM reported. *p <0.05, **p<0.01.

As a whole group overall, NAc ChR2 rats touched the *Laser-spout* >300% more (28.0±6.2 touches) than the *Inactive-spout* (8.7±2.7) across the 3 self-stimulation task days (*F*_1,6_ = 10.866, *p* = 0.016; Fig 7A). In contrast NAc eYFP rats (n = 6) touched equally between spouts across the 3 days of testing, significantly differing from NAc ChR2 rats (*F*_1,11_ = 7.289, *p* = 0.021). Individually, five out of seven NAc ChR2 rats met the lesser ≥10 *Laser-spout* criterion for low self-stimulation on day 1. The remaining 2 NAc ChR2 rats failed to self-stimulate by either criterion. The 5 NAc ChR2 rats classified as self-stimulators continued to self-stimulate at moderate levels on days 2–3, making *Laser-spout* 39.8 ± 5.2 touches, or >400% more than their *Inactive-spout* contacts (9.9±4.9; n = 5; *F*_1,4_ = 22.305, *p* = 0.009; Fig 7A). The remaining 2 NAc ChR2 individuals continued to fail to meet self-stimulation criteria on days 2–3.

#### CeA: Moderate CRF neuronal self-stimulation

As a group overall, CeA ChR2 rats showed a trend toward low rates of self-stimulation, making 37.4±9.4 *Laser-spout* touches versus 14.8±7.1 *Inactive-spout* touches (n = 8; *F*_1,7_ = 4.676, *p* = 0.067; Fig 7B). In contrast, control CeA eYFP rats (n = 6) made similar *Laser-spout* and *Inactive-spout* touches throughout the 3 test days (n = 6; *F*_1,5_ = 0.267, *p* = 0.628).

Individually, two of eight CeA ChR2 rats met the criterion for robust self-stimulation on day 1, while four met the criterion for low self-stimulation. Two CeA ChR2 rats failed to meet any self-stimulation criteria on day 1. On days 2–3, the six CeA ChR2 self-stimulators continued to self-stimulate at moderate rates of 40.7±10.6 laser illuminations per session versus 7.2±2.3 *Inactive-spout* touches (n = 6; *F*_1,5_ = 11.989, *p* = 0.018; Fig 7B), whereas the remaining two continued to fail to self-stimulate by either criterion.

#### BNST: No self-stimulation

As a group overall, BNST ChR2 rats failed to self-stimulate CRF-containing neurons by any criteria. Individually as well, all BNST ChR2 rats failed to meet criteria for laser self-stimulation, touching the *Laser-spout* (23.0±6.9) and *Inactive-spout* (23.6±6.4) roughly equally (n = 8; *F*_1,7_ = 0.000, *p* = 0.991; Fig 7C). BNST eYFP controls also responded similarly between spouts (n = 7; *F*_1,6_ = 2.355, *p* = 0.176).

#### Does laser self-stimulation correlate with increased cocaine pursuit?

Did NAc and CeA control of cocaine pursuit simply reflect a sum of laser value added to cocaine value? Or does NAc ChR2 and CeA ChR2 stimulation directly transform and amplify the value of paired cocaine, independently of laser value on its own? To assess these alternatives, we measured correlations between each individual’s laser self-stimulation and laser-paired cocaine pursuit in rats classified as self-stimulators.

**Fig 8:**
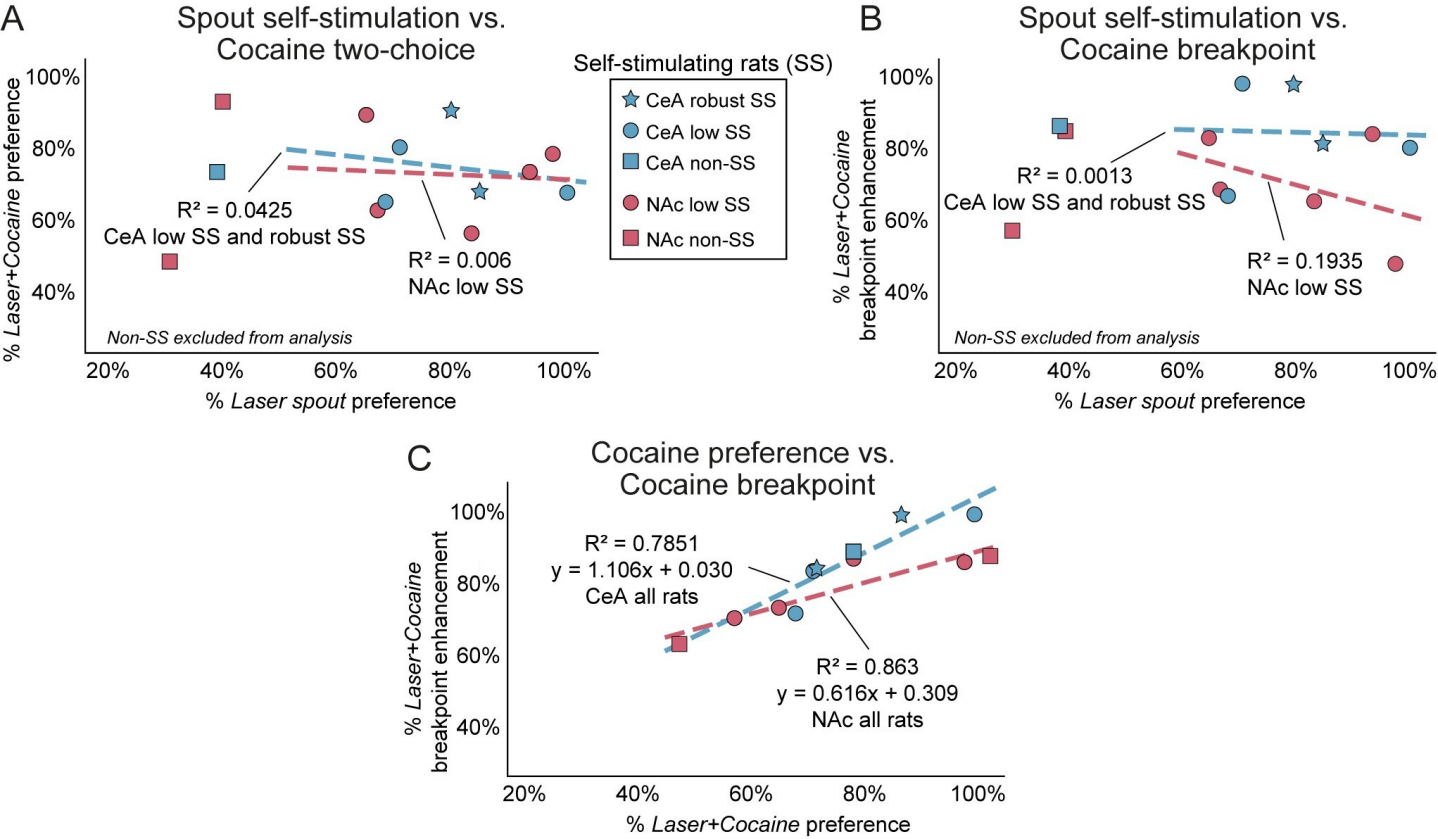
Laser-paired cocaine preference predicts cocaine breakpoint, not self-stimulation in NAc and CeA ChR2 rats. Laser self-stimulation by NAc ChR2 rats (red/purple squares & dashed line) or CeA ChR2 rats (blue squares & dashed line) did not correlate with their degree of laser-induced amplification of cocaine value. **A)** Lack of correlation between self-stimulation and paired cocaine preference in two-choice task. **B)** Lack of correlation between self-stimulation and intensity of paired cocaine motivation in breakpoint task. Even rats that met criteria for laser self-stimulation failed to show correlation with strength of laser-paired cocaine preference or pursuit. **C)** The magnitude of laser-induced increase in cocaine value was stable across both progressive ratio and two-choice tasks for NAc ChR2 and CeA ChR2 rats, as indicated by a simple linear regression analysis. Regression lines with β-coefficients and *R*^2^ values depicted.

Correlation results showed that laser self-stimulation levels failed to correlate or explain pursuit of paired *Laser+Cocaine* option in the two-choice task for either NAc ChR2 rats (n = 5, *r* = -0.078, *p* = 0.905) or CeA ChR2 rats (n = 5, *r* = -0.206, *p* = 0.739; Fig 8A). Similarly, laser self-stimulation failed to correlate with cocaine breakpoint increases in the progressive ratio task for either NAc ChR2 rats (*r* = -0.440, *p* = 0.459) or CeA ChR2 rats (*r* = 0.036, *p* = 0.955; Fig 8B). For example, individual NAc or CeA ChR2 rats that failed to meet any criteria for laser self-stimulation still showed 5:1 increases laser-paired cocaine pursuit and a laser-induced doubling of cocaine breakpoint, which were as high in magnitude as those of laser self-stimulators (Fig 8A and 8B). However, laser-paired cocaine preference was found to positively predict laser-based enhancement of cocaine breakpoint using a simple linear regression for both NAc ChR2 rats (*F*_1,5_ = 25.194, *p* = 0.007; *R*^*2*^ = 0.863) and CeA ChR2 rats (*F*_1,5_ = 14.612, *p* = 0.019; *R*^*2*^ = 0.785; Fig 8C), indicating that the degree of laser-induced amplification of paired cocaine value was reliably similar across both paradigms.

### Unilateral vs bilateral CRF ChR2 stimulation: Both behaviorally effective

CeA ChR2 rats with bilateral fiber/virus placements in CeA sites (n = 4) showed a 4:1 preference for their *Laser+Cocaine* option over the *Cocaine Alone* option. By comparison, CeA ChR2 rats with unilateral fiber/virus sites in CeA (n = 2; with contralateral sites were in the optic tract or basolateral amygdala) showed a nearly equivalent 3:1 preference for *Laser+Cocaine* option over *Cocaine alone* option. While a quantitative assessment would require greater N’s, we conclude that unilateral CeA CRF neuronal stimulation is sufficient to induce a preference for laser-paired cocaine. In the progressive ratio task, bilateral CeA ChR2 stimulation increased breakpoint for cocaine by 250% over the same rats’ breakpoint in absence of laser, and unilateral CeA ChR2 rats’ stimulation similarly increased breakpoint on laser day by 250% over baseline breakpoint in absence of laser. Again, unilateral and bilateral CeA CRF neuronal stimulation appear roughly comparable in enhancing motivation to consume cocaine. In the laser self-stimulation task, CeA ChR2 unilateral rats showed if anything stronger laser self-stimulation (n = 4, Laser illuminations: 42.9±14.0, Inactive spout touches: 7.8±10.0) than bilateral CeA ChR2 rats (n = 4, Laser illuminations: 31.9±14.0, Inactive spout touches: 21.7±10.0). Again, while quantitative assessment would require larger groups, we conclude that unilateral and bilateral CeA CRF neuronal excitations are both sufficient to support CRF neuronal self-stimulation behavior.

NAc ChR2 rats with bilateral sites in NAc shell (n = 3) showed stronger laser-induced preferences of 5:1 for their *Laser+Cocaine* option over their *Cocaine Alone* option in the 2-choice task compared to unilateral NAc ChR2 rats n = 4) that showed only a 2.5:1 preference for or Laser + Cocaine option over Cocaine alone option. Bilateral NAc ChR2 rats similarly showed higher 300% enhancements of breakpoint on *Laser+Cocaine* day compared to no-laser breakpoint, whereas unilateral rats showed only a 150% elevation of breakpoint on *Laser+Cocaine* day over no-laser breakpoint. We conclude that bilateral NAc CRF neuronal stimulation may well be more potent at enhancing incentive motivation for cocaine than unilateral stimulation. However, it appears that even unilateral NAc CRF neuronal stimulation may be sufficient enhance incentive motivation for cocaine over baseline levels. In the laser self-stimulation task, unilateral NAc ChR2 rats (n = 4) showed nearly identical levels of behavioral NAc CRF self-stimulation as bilateral NAc ChR2 rats (n = 3; Bilateral Laser illuminations: 29.0±10.4, Inactive spout touches: 11.4±4.2; Unilateral Laser illuminations: 27.2±9.0, Inactive spout touches: 6.6±3.6). This supports a conclusion that even unilateral stimulation of CRF neurons in NAc shell is sufficient to produce detectable incentive effects on behavior.

Neurobiologically, regarding Fos recruitment in distant mesolimbic structures, unilateral ChR2 CRF neuronal stimulation in rats that had only one fiber/virus site within their target structure (and a contralateral site instead in an adjacent structure) still evinced recruitment of increases in distant Fos expression in mesocorticolimbic structures. In only one distant structure (infralimbic cortex following BNST CRF stimulation) was unilateral-evoked Fos lower than bilateral-evoked Fos. Conversely, for NAc CRF stimulations, distant Fos in VTA and substantia nigra was actually higher after unilateral NAc stimulation than after bilateral stimulation. For most other structure/stimulation combinations, increases in Fos were evoked by both unilateral and bilateral CRF neuronal stimulations. We conclude overall that unilateral CRF neuronal stimulation in CeA, NAc and BNST may be sufficient to recruit mescorticolimbic Fos activations, though there may be moderate quantitative differences in a few cases. For this reason, both bilateral and unilateral rats are included in behavioral analyses above, and any differences noted when detected.

### Lack of overall sex differences in CRF ChR2 effects

Although moderate sex differences have been reported in cocaine self-administration and in CRF system anatomy and effects, there were no detectable sex differences here in overall cocaine intake of females (n = 21) and males (n = 16) over days in the two-choice task (*F*_1,29_ = 0.123, *p* = 0.729). Females and males also consumed similar amounts of cocaine overall in the progressive ratio task (female n = 21; male n = 15; *F*_1,30_ = 0.321, *p* = 0.575). There were also no significant interactions between sex and laser conditions or limbic sites overall, although we note that N’s became too small to detect potential sex differences in the relative intensity of effects for specific site/experiment/laser combinations, especially if any quantitative sex differences are small to moderate in size. Given the overall comparable cocaine pursuit of both sexes here, females and males were analyzed together except where explicitly stated.

Similarly, regarding laser self-stimulation of CRF neurons, there was no overall sex difference between female and male C*rh-*Cre rats with ChR2 virus in laser self-stimulation patterns (females n = 22, males n = 14; *F*_1,33_ = 0.528, *p* = 0.473). We caution that both for cocaine self-administration and laser self-stimulation, the numbers of females versus males in specific anatomical site groups were not sufficiently powered to rule out the possibility of slight quantitative sex differences. However, overall similarity in qualitative effects here suggests that females and males do not at least differ categorically in propensity for CRF neuronal laser self-stimulation or in optogenetic CRF neuronal effects on motivation for cocaine.

## Discussion

Pairing optogenetic excitation of CRF-containing neurons in NAc or CeA with a particular i.v. cocaine reward in *Crh*-Cre rats doubled the intensity of pursuit and consumption of the laser-paired cocaine option, while the same rats relatively neglected their alternative option of cocaine without laser. Laser excitation of CRF-containing neurons in NAc or CeA caused: 1) an overall 4:1 preference for the *Laser+Cocaine* option over the equal *Cocaine-alone* option by the end of testing, narrowing the focus of incentive motivation; 2) increases in the *amplitude* of their incentive motivation for laser-paired cocaine, expressed as a doubling of breakpoint in the progressive ratio task, as well as by escalated cocaine consumption in the two-choice task; and 3) a slight but detectable positive motivational valence of CRF-containing neuronal activation itself, revealed as low to moderate levels of laser self-stimulation by most CeA and NAc ChR2 rats. Evidence that increased recruitment of mesocorticolimbic circuitry mediated the enhancement of incentive motivation was provided by observing increases of neuronal Fos activation in VTA, NAc, VP, etc after laser ChR2 stimulations of CRF neurons in either CeA or NAc.

Our results demonstrate for the first time that stimulating CRF-containing neurons in either NAc or CeA can promote choice, pursuit, and consumption of a drug such as cocaine via a positively-valenced incentive motivation process, just as similar CRF neuronal stimulation promotes incentive motivation for sucrose reward [19].

### CRF peptide versus co-released neurotransmitters?

We emphasize that our current study is meant to examine the roles of CRF *neurons* in motivation for cocaine, a relatively unstudied aspect of CRF motivational systems. Our results do not make claims for the role of CRF peptide specifically as a neurotransmitter, or for roles of particular CRF neurochemical receptors. That is because CRF-expressing neurons, in addition to releasing CRF peptide, also co-release several other neurotransmitters, including GABA, glutamate, dynorphin, neurotensin, and somatostatin [28–31]. Future research could examine which of these neurochemical signals released by CRF-containing neurons in NAc or CeA are most responsible for amplifying ‘wanting’ of cocaine rewards. Conceivably, a contributing role for at least some co-released neurotransmitters could be related to why CRFR1 antagonists sometimes fail to block stress-induced craving in clinical studies [32–35].

### Contributions of CRF neuronal projections?

Future research could also identify which CRF-releasing anatomical projections mediate incentive motivation for cocaine. CRF-containing neurons in CeA project to downstream targets in LH, VP, VTA, PBN and BNST [23, 30, 36–38]. Projections to LH, VP, or VTA may be primary candidates to mediate incentive motivation, as stimulation of CeA-BNST projections are reliably reported to induce negatively-valenced motivation [37, 39–41]. In NAc medial shell, CRF-containing neurons may make mostly local connections within NAc, including to cholinergic interneurons that may modulate local dopamine release [15, 42].

### CRF-containing neurons in BNST lack incentive roles

In contrast to most NAc ChR2 and CeA ChR2 rats, BNST ChR2 rats uniformly failed to self-stimulate laser here, and excitation of CRF-containing neurons in BNST failed to increase cocaine pursuit. Instead, activation of CRF-containing neurons in BNST prevented the usual escalation of cocaine intake that otherwise occurred over multiple days in the two-choice task, consequently reducing final cocaine intake in comparison to NAc and CeA ChR2 rats. In the progressive ratio task, stimulation of CRF-containing neurons in BNST failed to alter cocaine breakpoint, somewhat different than in our previous study where BNST stimulation reduced sucrose breakpoint [19]. Also, stimulation of CRF-containing neurons in BNST failed to alter cocaine preference here in the two-choice task, whereas it caused avoidance of the laser-paired sucrose reward in our previous study. These slight BNST differences in cocaine vs sucrose results between the two studies might reflect group differences or procedural differences between the two studies (e.g., single-housing and food restriction was used for cocaine groups but not sucrose groups), or reflect unique features of cocaine that are not shared by sucrose as rewards. However, BNST CRF-containing neuron excitation here did confirm previously found recruitment of Fos activation in downstream structures related to stress or pain, including PAG and PVN [19].

### Laser CRF self-stimulation is insufficient to account for cocaine pursuit

Approximately ~75%-80% of NAc ChR2 rats and CeA ChR2 rats self-stimulated for laser illumination at least at low to moderate levels, implying that CRF-containing neuronal excitation in NAc and CeA carried positive incentive value [17, 19]. That raises the question of whether NAc ChR2 and CeA ChR2 rats preferred their *Laser+Cocaine* option in the two-choice task because it simply combined the sum of two separate incentives? We believe that is unlikely, because the 20% of NAc ChR2 and CeA ChR2 individuals that failed to self-stimulate at all still showed laser-induced pursuit of *Laser+Cocaine* comparable to other rats that did self-stimulate for laser excitations of their CRF neurons. Further, the intensity of laser self-stimulation across rats that did self-stimulate failed to correlate with their intensity of *Laser+Cocaine* pursuit.

Instead, we suggest that CRF-containing neuronal activation in NAc or CeA directly transformed the incentive value of laser-paired cocaine in a multiplicative manner, making that particular option more intensely ‘wanted’. Thus, the incentive salience value of laser-paired cocaine became more than the sum of its parts [19, 22, 25, 43].

That incentive transformation hypothesis is similar to the interpretation of previous optogenetic CeA studies that induced intense, focused pursuit of laser-paired cocaine, sucrose, or even a shock-rod [19, 22, 25, 43]. Such affectively potent stimuli may serve as better targets for optogenetic attribution of intense incentive salience than neutral stimuli (i.e., empty spouts for self-stimulation) because they themselves elicit emotional reactions and mesocorticolimbic activations, making them able to serve effectively as unconditioned stimuli in Pavlovian paradigms. We hypothesize that simultaneous laser excitation of CRF-containing systems in NAc or CeA, combined with cocaine-induced activation of mesocorticolimbic circuitry, powerfully amplifies the magnitude of incentive salience assigned to cues for the laser-paired cocaine. This makes that particular cocaine option and its cues become intensely ‘wanted’. Such transformation of laser-paired cocaine value may explain why excitation of CRF-containing neurons in CeA or NAc can create a narrowly-focused motivation for cocaine, yet the laser remains a relatively weak reinforcer by itself in self-stimulation tasks.

### Implications for addiction neuroscience

CRF-containing neurons in CeA and BNST have been implicated in distressing feelings of drug withdrawal and anxiety-like states [5, 44–46]. Evidence suggests that CRF systems neurons in CeA and BNST also contribute to anxiety and fear learning [24, 28, 37, 40, 47–51]. Such findings have fueled assumptions in neuroscience research that activation of CRF-containing neurons is intrinsically aversive.

Our results support an alternative hypothesis for how activation of CRF-containing neurons, at least in NAc and CeA, can promote pursuit and consumption of cocaine and related rewards via a positively-valenced motivation process, such as incentive salience or ‘wanting’. This incentive motivation hypothesis provides an alternative way of understanding how CRF-containing neurons activated by stress may increase in pursuit and consumption of rewards. Our findings add to the growing body of literature supporting a range of flexibly-valenced motivational roles for CRF systems, capable of initiating bio-behavioral responses to both positive and negative stimuli [10, 11, 15–17, 42, 52].

### Valence flexibility of CRF neurons?

We acknowledge that the motivational valence of CRF neuronal excitation in NAc or CeA might conceivably flip in other circumstances from a positively-valenced incentive motivation role to the opposite negatively-valenced or aversive role that has been traditionally posited. For example, diverse aversive states including withdrawal following chronic drug use, such as those modeled by long-access procedures for cocaine-self administration, might conceivably flip CeA or NAc CRF-containing neurons to an aversive role, or endow BNST CRF-containing neurons with a novel ability to increase cocaine pursuit [7, 8, 53, 54]. Such valence reversals might occur under diverse motivational conditions, as CRF neural signals can flexibly initiate bio-behavioral responses to both positive incentives and negative stressors [52].

## Conclusion

Understanding the role of CRF-containing neurons in motivation will be integral to understanding potential contributions to mood disorders and addictive relapse. Our results suggest that CRF-containing neurons in either NAc shell or CeA can cause intense pursuit and increased consumption of cocaine through a positively-valenced motivational process, such as incentive salience or ‘wanting’ for drugs. This is similar to incentive roles of CRF-containing neurons in NAc and CeA that promote sucrose pursuit [14, 16, 19], and is compatible with suggestions that CRF system activation promotes over-eating of “comfort foods” primarily via positively-valenced processes that increase ‘wanting’ for those foods [55].

Incentive roles of CRF-containing neurons may help explain why relapse in drug pursuit and addiction and binge eating can be triggered by positively-valenced stressors, such as forming a new romantic relationship or winning the lottery [56–61]. Incentive motivation roles of CRF-containing neurons may also help explain why vulnerability to relapse in addiction persists long after aversive withdrawal feelings and other life distresses subside [62, 63]. Ultimately, the multiple motivational roles of CRF neural systems may be crucial to understanding the brain mechanisms underlying mood disorders as well as addictive relapse and substance abuse [16].

## Supporting information

S1 File (DOCX)
