# Supplementary material for: Corticotropin releasing factor (CRF) systems: Promoting cocaine pursuit without distress via incentive motivation
Source: PLoS One. 2022 May 3;17(5):e0267345. doi: 10.1371/journal.pone.0267345 (PMC9064096; doi:10.1371/journal.pone.0267345)

**Supporting Information**

*Corticotropin releasing factor (CRF) systems: promoting cocaine pursuit without distress via incentive motivation*

Baumgartner, Granillo, Schulkin, & Berridge (2022)

| **Target** | **Confirmed placement ranges (mm from Bregma)** | | | **ChR2**  **N’s** | | | | **eYFP**  **N’s** | | | | Contralateral misses, locations |
| --- | --- | --- | --- | --- | --- | --- | --- | --- | --- | --- | --- | --- |
|  | A/P | M/L | D/V | Uni | | Bil | | Uni | | Bil | |  |
|  |  |  |  | F | M | F | M | F | M | F | M |  |
| NAc | +2.52 to 1.08 | ± 0.6 to 1.8 | -6.0 to  -7.2 | 3 | 1 | 2 | 1 | 3 | 0 | 2 | 1 | NAcC, VP, DS |
|  |  |  |  |  |  |  |  |  |  |  |  |  |
| CeA | -1.92 to  -3.24 | ± 3.6 to 4.6 | -6.8 to  -8.4 | 1 | 2 | 3 | 1 | 1 | 2 | 2 | 1 | MeA, optic tract, BLA |
| BNST | +0.36 to  -0.36 | ±1.4 to 1.8 | -6.0 to  -6.5 | 1 | 3 | 2 | 3 | 1 | 3 | 2 | 2 | LS, ac |

**Table S1. Histological placements of experimental animals**

Table displays all experiment animals, broken up by target region, sex (F, female; M, male), virus (ChR2/eYFP) and whether they were confirmed as having bilaterally (bil) correct histological placements or a unilateral (uni) correct placement. Coordinates and N’s of ChR2 and eYFP *Crh*-Cre+ rats for central amygdala (CeA), nucleus accumbens (NAc), and bed nucleus of stria terminalis (BNST) target groups. The far-right column displays locations where contralaterally missed placements for unilateral rats were confirmed. MeA, medial amygdala; BLA, basolateral amygdala; NAcC, nucleus accumbens core; VP, ventral pallidum; DS, dorsal striatum; LS, lateral septum; ac, anterior commissure.

| NAc CRF neurons | Fos+ count  Mean ± SEM | | t-statistic, p-value | |  |
| --- | --- | --- | --- | --- | --- |
| Region | NAc ChR2 Unilateral (n=4) | NAc ChR2 Bilateral (n=3) | *t* | *p* | |
| IF | 22.7 ± 1.9 | 22.3 ± 3.8 | 0.078 | *0.942* | |
| OFC | 56.0 ± 7.0 | 54.7 ± 10.6 | 0.105 | *0.921* | |
| NAcC | 76.7 ± 10.4 | 62.7 ± 5.4 | 1.197 | *0.298* | |
| aVP | 55.3 ± 1.5 | 50.7 ± 1.5 | 2.271 | *0.086* | |
| pVP | 53.7 ± 4.9 | 47.7 ± 2.9 | 1.052 | *0.352* | |
| aBNST | 36.0 ± 4.8 | 44.7 ± 6.4 | 1.094 | *0.335* | |
| pBNST | 60.7 ± 9.8 | 56.7 ± 5.2 | 0.360 | *0.737* | |
| aLH | 47.0 ± 9.8 | 45.3 ± 5.0 | 0.151 | *0.888* | |
| pLH | 49.3 ± 0.9 | 42.7 ± 3.7 | 1.747 | *0.155* | |
| PVN | 38.0 ± 7.8 | 36.7 ± 2.8 | 0.161 | *0.880* | |
| MeA | 57.3 ± 1.8 | 45.7 ± 10.3 | 1.120 | *0.326* | |
| CeA | 33.0 ± 0.6 | 39.0 ± 4.0 | 1.470 | *0.216* | |
| BLA | 29.0 ± 1.5 | 36.3 ± 5.2 | 1.344 | *0.250* | |
| VTA | 64.3 ± 1.5 | 42.0 ± 4.9 | 4.343 | *0.012** | |
| SN | 24.0 ± 0.6 | 19 ± 1.0 | 4.330 | *0.012** | |
| PAG | 27.3 ± 4.8 | 30.3 ± 6.7 | 0.364 | *0.734* | |

**Table S2. Unilateral vs. bilateral brain-wide Fos activation following CRF-containing neuron excitation NAc.** Table shows Fos+ protein quantification in mesocorticolimbic regions after final exposure to ChR2 excitation in rats with either unilateral (n=3) or bilateral (n=3) optic fiber placements within the NAc (n=3 female, n=3 male ChR2). Fos+ protein quantification in mesocorticolimbic regions (left columns) for ChR2 rats with unilaterally versus bilaterally correct optic fiber placements were compared with a one-way ANOVA for each structure targeted. “Fos+ Count” reflects the mean of each group at each site ± standard error (SEM). Indepent *t*-test values and two-tailed *p* values are reported in the right column.Percent change in ChR2 rats from eYFP controls are depicted in Fig. 3 and Table 1. IF, infralimbic cortex; OFC, orbitofrontal cortex; aNAcSh, anterior nucleus accumbens shell; pNAcSh, posterior nucleus accumbens shell; NAcC, nucleus accumbens core; aVP, anterior ventral pallidum; pVP, posterior ventral pallidum; aBNST, anterior bed nucleus of stria terminalis; pBNST, posterior bed nucleus of stria terminalis; aLH, anterior lateral hypothalamus; pLH, posterior lateral hypothalamus; PVN, hypothalamic paraventricular nucleus; MeA, medial amygdala; CeA, central amygdala; BLA, basolateral amygdala; VTA, ventral tegmentum; SN, substantia nigra; PAG, midbrain periaqueductal gray. *p<0.05, **p<0.01, ***p<0.001

| CeA CRF neurons | Fos+ count  Mean ± SEM | | t-statistic, p-value | | |
| --- | --- | --- | --- | --- | --- |
| Region | CeA ChR2 Unilateral (n=3) | CeA ChR2 Bilateral (n=4) | | *t* | *p* |
| IF | 18.5 ± 3.5 | 25.0 ± 1.6 | | 2.033 | *0.112* |
| OFC | 51.0 ± 19.0 | 54.3 ± 5.7 | | 0.225 | *0.833* |
| NAcC | 86.5 ± 19.5 | 81.3 ± 8.0 | | 0.309 | *0.773* |
| aNAcSh | 49.5 ± 8.5 | 58.8 ± 7.7 | | 0.734 | *0.504* |
| pNAcSh | 79.0 ± 2.0 | 91.0 ± 5.4 | | 0.396 | *0.214* |
| aVP | 51.0 ± 5.0 | 49.8 ± 7.7 | | 0.105 | *0.922* |
| pVP | 58.0± 6.0 | 54.5 ± 6.0 | | 0.361 | *0.736* |
| aBNST | 47.0 ± 7.0 | 41.8 ± 7.3 | | 0.447 | *0.678* |
| pBNST | 62.0 ± 7.0 | 59.3 ± 7.0 | | 0.242 | *0.821* |
| aLH | 43.0 ± 4.0 | 46.5 ± 3.1 | | 0.671 | *0.539* |
| pLH | 37.0 ± 10.0 | 49.5 ± 3.2 | | 1.611 | *0.182* |
| PVN | 32.0 ± 5.0 | 37.5 ± 3.5 | | 0.900 | *0.419* |
| MeA | 42.0 ± 6.0 | 47.0 ± 2.6 | | 0.930 | *0.405* |
| BLA | 43.5 ± 0.5 | 37.0 ± 6.8 | | 0.637 | *0.558* |
| VTA | 49.0 ± 3.0 | 40.5 ± 6.3 | | 0.877 | *0.430* |
| SN | 30.0 ± 13.0 | 23.3 ± 3.0 | | 0.741 | *0.500* |
| PAG | 31.5 ± 2.5 | 33.8 ± 5.0 | | 0.294 | *0.784* |

**Table S3. Unilateral vs. bilateral brain-wide Fos activation following CRF-containing neuron excitation CeA.** Table shows Fos+ protein quantification in mesocorticolimbic regions after final exposure to ChR2 excitation in rats with either unilateral (n=2) or bilateral (n=4) optic fiber placements within the CeA (n=3 female, n=3 male ChR2). Fos+ protein quantification in mesocorticolimbic regions (left columns) for ChR2 rats with unilaterally versus bilaterally (n=4) correct optic fiber placements were compared with a one-way ANOVA for each structure targeted. “Fos+ Count” reflects the mean of each group at each site ± standard error (SEM). Indepent *t*-test values and two-tailed *p-*values are reported in the right column. Percent change in ChR2 rats from eYFP controls are depicted in Fig. 3 and Table 2. IF, infralimbic cortex; OFC, orbitofrontal cortex; aNAcSh, anterior nucleus accumbens shell; pNAcSh, posterior nucleus accumbens shell; NAcC, nucleus accumbens core; aVP, anterior ventral pallidum; pVP, posterior ventral pallidum; aBNST, anterior bed nucleus of stria terminalis; pBNST, posterior bed nucleus of stria terminalis; aLH, anterior lateral hypothalamus; pLH, posterior lateral hypothalamus; PVN, hypothalamic paraventricular nucleus; MeA, medial amygdala; CeA, central amygdala; BLA, basolateral amygdala; VTA, ventral tegmentum; SN, substantia nigra; PAG, midbrain periaqueductal gray. *p<0.05

| BNST CRF neurons | Fos+ count  Mean ± SEM | | | t-statistic, p-value | | |
| --- | --- | --- | --- | --- | --- | --- |
| Region | BNST ChR2 Unilateral (n=4) | BNST ChR2 Bilateral (n=5) | *t* | | *p* |  |
| IF | 11.0 ± 1.0 | 20.3 ± 1.4 | 4.294 | | *0.013** |  |
| OFC | 39.0 ± 8.0 | 59.0 ± 11.3 | 1.132 | | *0.321* |  |
| NAcC | 30.0 ± 4.0 | 58.3 ± 10.2 | 1.819 | | *0.143* |  |
| aNAcSh | 23.5 ± 5.5 | 41.5 ± 4.3 | 2.495 | | *0.067* |  |
| pNAcSh | 38.5 ± 3.5 | 52.3 ± 5.0 | 1.772 | | *0.151* |  |
| aVP | 26.5 ± 0.5 | 28.0 ± 5.1 | 0.196 | | *0.854* |  |
| pVP | 30.5 ± 3.5 | 42.0 ± 9.4 | 1.591 | | *0.187* |  |
| aLH | 29.0 ± 5.0 | 30.0 ± 1.1 | 0.289 | | *0.787* |  |
| pLH | 32.5 ± 1.5 | 36.0 ± 5.7 | 0.406 | | *0.706* |  |
| PVN | 33.5 ± 3.5 | 35.0 ± 2.3 | 0.364 | | *0.734* |  |
| MeA | 31.5 ± 2.5 | 35.0 ± 5.6 | 0.409 | | *0.703* |  |
| CeA | 22.5 ± 5.5 | 19.3 ± 2.8 | 0.606 | | *0.577* |  |
| BLA | 43.0 ± 14.0 | 35.5 ± 8.7 | 0.479 | | *0.657* |  |
| VTA | 39.5 ± 9.5 | 33.0 ± 3.7 | 0.844 | | *0.446* |  |
| SN | 20.5 ± 1.5 | 22.3 ± 2.0 | 0.554 | | *0.609* |  |
| PAG | 28.0 ± 0.0 | 32.8 ± 1.8 | 1.810 | | *0.145* |  |

**Table S4. Unilateral vs. bilateral brain-wide Fos activation following CRF-containing neuron excitation BNST.** Table shows Fos+ protein quantification in mesocorticolimbic regions after final exposure to ChR2 excitation in rats with either unilateral (n=2) or bilateral (n=4) optic fiber placements within the BNST (n=3 female, n=3 male ChR2). Fos+ protein quantification in mesocorticolimbic regions (left columns) for ChR2 rats with unilaterally versus bilaterally (n=4) correct optic fiber placements were compared with a one-way ANOVA for each structure targeted. “Fos+ Count” reflects the mean of each group at each site ± standard error (SEM). Indepent *t*-test values and two-tailed *p* values are reported in the right column. Percent change in ChR2 rats from eYFP controls are depicted in Fig. 3 and Table 3. IF, infralimbic cortex; OFC, orbitofrontal cortex; aNAcSh, anterior nucleus accumbens shell; pNAcSh, posterior nucleus accumbens shell; NAcC, nucleus accumbens core; aVP, anterior ventral pallidum; pVP, posterior ventral pallidum; aBNST, anterior bed nucleus of stria terminalis; pBNST, posterior bed nucleus of stria terminalis; aLH, anterior lateral hypothalamus; pLH, posterior lateral hypothalamus; PVN, hypothalamic paraventricular nucleus; MeA, medial amygdala; CeA, central amygdala; BLA, basolateral amygdala; VTA, ventral tegmentum; SN, substantia nigra; PAG, midbrain periaqueductal gray. *p<0.05

| Two-choice Cocaine Self-Administration  Responses | | **ANOVA**  **df, F-statistic, p-value** | | |
| --- | --- | --- | --- | --- |
| Group | Effect | *df* | *F* | *p* |
| NAc ChR2  N = 7 | Day | *--* | -- | *--* |
|  | Laser | *1, 6* | 6.970 | *0.039** |
|  | Day x Laser | *--* | -- | *--* |
| NAc eYFP  N = 6 | Day | *--* | -- | *--* |
|  | Laser | *1, 5* | 1.367 | *0.295* |
|  | Day x Laser | *--* | -- | *--* |
| NAc  ChR2 vs. eYFP | Day | *9, 3* | 2.797 | *0.215* |
|  | Day x Virus | *9, 3* | 0.589 | *0.763* |
|  | Laser | *1, 11* | 7.485 | *0.019** |
|  | Laser x Virus | *1, 11* | 1.988 | *0.186* |
|  | Day x Laser | *9, 3* | 28.595 | *0.009*** |
|  | Day x Laser x Virus | *9, 3* | 12.320 | *0.031** |
|  | Virus | *1, 11* | 1.333 | *0.273* |
| CeA ChR2  N = 6 | Day | *--* | -- | *--* |
|  | Laser | *1, 5* | 14.669 | *0.012** |
|  | Day x Laser | *--* | -- | *--* |
| CeA eYFP  N = 6 | Day | *--* | -- | *--* |
|  | Laser | *1,5* | 0.540 | *0.495* |
|  | Day x Laser | *--* | -- | *--* |
| CeA  ChR2 vs. eYFP | Day | *9, 2* | 1.023 | *0.587* |
|  | Day x Virus | *9, 2* | 0.583 | *0.766* |
|  | Laser | *1, 10* | 15.068 | *0.003*** |
|  | Laser x Virus | *1, 10* | 12.021 | *0.006*** |
|  | Day x Laser | *9, 2* | 3.385 | *0.249* |
|  | Day x Laser x Virus | *9, 2* | 0.502 | *0.808* |
|  | Virus | *1, 10* | 7.051 | *0.024** |
| BNST ChR2  N = 6 | Day | *--* | -- | *--* |
|  | Laser | *1, 5* | 0.000 | *1.000* |
|  | Day x Laser | *--* | -- | *--* |
| BNST eYFP  N =6 | Day | *--* | -- | *--* |
|  | Laser | *1, 5* | 3.773 | *0.110* |
|  | Day x Laser | *--* | -- | *--* |
| BNST  ChR2 vs. eYFP | Day | *9, 2* | 0.849 | *0.649* |
|  | Day x Virus | *9, 2* | 1.419 | *0.480* |
|  | Laser | *1, 10* | 1.398 | *0.264* |
|  | Laser x Virus | *1, 10* | 1.398 | *0.264* |
|  | Day x Laser | *9, 2* | 0.649 | *0.734* |
|  | Day x Laser x Virus | *9, 2* | 63.637 | *0.016** |
|  | Virus | *1, 10* | 1.590 | *0.236* |

**Table S5. Two-way ANOVA statistics for number of responses for cocaine in the two-choice task.** The number of responses for either *Laser-paired* or *Cocaine alone* options in the two-choice self-administration test were compared across the 10 test days for ChR2 and eYFP groups targeting either the nucleus accumbens (NAc), central amygdala (CeA), and the bed nucleus of the stria terminalis (BNST). The first column describes the group being analyzed in the two-way ANOVA. The second column shows effects tested including the main effect of day (i.e., across 10 test days), effect of laser (i.e., whether the response was for the laser-paired option or non-laser paired option), effect of virus (i.e., ChR2 or eYFP groups), and interactions between these effects. Degrees of freedom (df), F-statistics (*F*), and two-tailed p-values (*p*) for each effect are listed in the right columns. *p<0.05, **p<0.01

| Two-choice Cocaine Self-Administration Infusions | | **ANOVA**  **df, F-statistic, p-value** | | |
| --- | --- | --- | --- | --- |
| Target | Effect | *df* | *F* | *p* |
| NAc ChR2  N = 7 | Day | *--* | -- | *--* |
|  | Laser | *1, 6* | 6.093 | *0.049** |
|  | Day x Laser | *--* | -- | *--* |
| NAc eYFP  N = 6 | Day | *--* | -- | *--* |
|  | Laser | *1, 5* | 2.344 | *0.299** |
|  | Day x Laser | *--* | -- | *--* |
| NAc  ChR2 vs. eYFP | Day | *9, 3* | 1.468 | *0.415* |
|  | Day x Virus | *9, 3* | 1.136 | *0.513* |
|  | Laser | *1, 11* | 6.737 | *0.025** |
|  | Laser x Virus | *1, 11* | 1.645 | *0.226* |
|  | Day x Laser | *9, 3* | 44.785 | *0.005*** |
|  | Day x Laser x Virus | *9, 3* | 18.366 | *0.018** |
|  | Virus | *1, 11* | 0.926 | *0.357* |
| CeA ChR2  N = 6 | Day | *--* | -- | *--* |
|  | Laser | *1, 5* | 13.506 | *0.014** |
|  | Day x Laser | *--* | -- | *--* |
| CeA eYFP  N = 6 | Day | *--* | -- | *--* |
|  | Laser | *1, 5* | 0.469 | *0.524* |
|  | Day x Laser | *--* | -- | *--* |
| CeA  ChR2 vs. eYFP | Day | *9, 2* | 1.390, | *0.487* |
|  | Day x Virus | *9, 2* | 0.396 | *0.865* |
|  | Laser | *1, 10* | 13.930 | *0.004** |
|  | Laser x Virus | *1, 10* | 1.594 | *0.007** |
|  | Day x Laser | *9, 2* | 1.384 | *0.488* |
|  | Day x Laser x Virus | *9, 2* | 0.474 | *0.823* |
|  | Virus | *1, 10* | 10.329 | *0.009*** |
| BNST ChR2  N = 6 | Day | *--* | -- | *--* |
|  | Laser | *1, 5* | 0.083 | *0.784* |
|  | Day x Laser | *--* | -- | *--* |
| BNST eYFP  N = 6 | Day | *--* | -- | *--* |
|  | Laser | *1, 5* | 6.853 | *0.047** |
|  | Day x Laser | *--* | -- | *--* |
| BNST  ChR2 = 6, eYFP = 6 | Day | *9, 2* | 2.364 | *0.333* |
|  | Day x Virus | *9, 2* | 1.268 | *0.517* |
|  | Laser | *1, 10* | 3.342 | *0.097* |
|  | Laser x Virus | *1, 10* | 1.880 | *0.200* |
|  | Day x Laser | *9, 2* | 0.691 | *0.715* |
|  | Day x Laser x Virus | *9, 2* | 7.038 | *0.131* |
|  | Virus | *1, 10* | 1.273 | *0.286* |

**Table S6. Two-way ANOVA statistics for number of cocaine infusions in the two-choice task.** The number of earned cocaine infusions for either *Laser-paired* or *Cocaine alone* options in the two-choice self-administration test were compared across the 10 test days for ChR2 and eYFP groups targeting either the nucleus accumbens (NAc), central amygdala (CeA), and the bed nucleus of the stria terminalis (BNST). The first column describes the group being analyzed in the two-way ANOVA. The second column shows effects tested including the main effect of day (i.e., across 10 test days), effect of laser (i.e., whether the cocaine infusion was the laser-paired option or the non-laser paired option), effect of virus (i.e., ChR2 or eYFP control groups), and interactions between these effects. Degrees of freedom (df), F-statistics (*F*), and two-tailed p-values (*p*) for each effect are listed in the right columns. *p<0.05, **p<0.01

| Laser self-stimulation  All rats – Test days 1-3 | | **ANOVA**  **df, F-statistic, p-value** | | |
| --- | --- | --- | --- | --- |
| Target | Effect | *df* | *F* | *p* |
| NAc ChR2  N = 7 | Day | *2, 5* | 2.393 | *0.187* |
|  | Laser | *1, 6* | 10.866 | *0.016** |
|  | Day x Laser | *2, 5* | 1.466 | *0.315* |
| NAc eYFP  N = 6 | Day | *2, 4* | 0.573 | *0.604* |
|  | Laser | *1, 5* | 0.034 | *0.861* |
|  | Day x Laser | *2, 4* | 1.689 | *0.294* |
| NAc  ChR2 vs. eYFP | Day | *2, 10* | 2.590 | *0.124* |
|  | Day x Virus | *2, 10* | 1.105 | *0.368* |
|  | Laser | *1, 11* | 8.178 | *0.016** |
|  | Laser x Virus | *1, 11* | 7.289 | *0.021** |
|  | Day x Laser | *2, 10* | 0.473 | *0.636* |
|  | Day x Laser x Virus | *2, 10* | 3.555 | *0.068* |
|  | Virus | *1, 11* | 4.345 | *0.061* |
| CeA ChR2  N = 8 | Day | *2, 6* | 0.274 | *0.769* |
|  | Laser | *1, 7* | 4.676 | *0.067* |
|  | Day x Laser | *2, 6* | 1.193 | *0.366* |
| CeA eYFP  N = 6 | Day | *2, 4* | 1.669 | *0.297* |
|  | Laser | *1, 5* | 0.267 | *0.628* |
|  | Day x Laser | *2, 4* | 0.532 | *0.624* |
| CeA  ChR2 vs. eYFP | Day | *2, 11* | 0.537 | *0.599* |
|  | Day x Virus | *2, 11* | 0.408 | *0.674* |
|  | Laser | *1, 12* | 1.913 | *0.192* |
|  | Laser x Virus | *1, 12* | 3.715 | *0.078* |
|  | Day x Laser | *2, 11* | 0.503 | *0.618* |
|  | Day x Laser x Virus | *2, 11* | 1.066 | *0.377* |
|  | Virus | *1, 12* | 3.132 | *0.102* |
| BNST ChR2  N = 8 | Day | *2, 6* | 1.570 | *0.283* |
|  | Laser | *1, 7* | 0.000 | *0.991* |
|  | Day x Laser | *2, 6* | 0.125 | *0.885* |
| BNST eYFP  N = 7 | Day | *2, 5* | 1.618 | *0.287* |
|  | Laser | *1, 6* | 2.355 | *0.176* |
|  | Day x Laser | *2, 5* | 0.480 | *0.644* |
| BNST  ChR2 vs. eYFP | Day | *2, 12* | 3.203 | *0.077* |
|  | Day x Virus | *2, 12* | 0.466 | *0.638* |
|  | Laser | *1, 13* | 0.181 | *0.677* |
|  | Laser x Virus | *1, 13* | 0.200 | *0.662* |
|  | Day x Laser | *2, 12* | 0.249 | *0.783* |
|  | Day x Laser x Virus | *2, 12* | 0.103 | *0.903* |
|  | Virus | *1, 13* | 1.606 | *0.227* |

**Table S7. Two-way ANOVA statistics for self-stimulation test days 1-3.** The number of interactions with either the *Laser-spout* or the *Inactive-spout* in the spout self-stimulation test were compared across the 3 test days for all NAc (n=7 ChR2, n=6 eYFP), CeA (n=8 ChR2, n=6 eYFP), and BNST (n=8 ChR2, n=7 eYFP) groups. The first column describes the group being analyzed in the two-way ANOVA. The second column shows the effects tested including the main effect of day (i.e., across 3 test days), effect of laser (i.e., whether the response was for the *Laser-spout* or *Inactive-spout*), effect of virus (i.e., ChR2 or eYFP groups), and interactions between these effects. Degrees of freedom (df), F-statistics (*F*), and two-tailed p-values (*p*) for each effect are listed in the right columns. *p<0.05

| Laser self-stimulation  Self-stimulators – Test days 2-3 | | **ANOVA**  **df, F-statistic, p-value** | | |
| --- | --- | --- | --- | --- |
| Target | Effect | *df* | *F* | *p* |
| NAc ChR2  Self-stimulators  N = 5 | Day | *1, 4* | *2.955* | *0.161* |
|  | Laser | *1, 4* | 22.305 | *0.009*** |
|  | Day x Laser | *1, 4* | 2.953 | *0.161* |
| CeA ChR2  Self-stimulators  N = 6 | Day | *1, 5* | 0.164 | *0.703* |
|  | Laser | *1, 5* | 11.989 | *0.018** |
|  | Day x Laser | *1, 5* | 0.295 | *0.611* |

**Table S8. Two-way ANOVA statistics for rats that met self-stimulation criteria.** The number of interactions with either the *Laser-spout* or the *Inactive-spout* in the spout self-stimulation test were compared across test days 2-3 for rats that met self-stimulation criteria on test day 1. The NAc group included 5 ChR2 rats that met criteria for low-level self-stimulation on day 1 and the CeA group included 4 rats that met criteria for low-level self-stimulation on day 1 and 2 more rats that met criteria for robust self-stimulation on day 1. All eYFP groups and the BNST ChR2 group did not have rats that met self-stimulation criteria on day 1, and therefore were not included. The first column describes the group being analyzed in the two-way ANOVA. The second column shows the effects tested including the main effect of day (i.e., across 3 test days), effect of laser (i.e., whether the response was for the *Laser-spout* or *Inactive-spout*), and the interaction between these effects. Degrees of freedom (df), F-statistics (*F*), and two-tailed p-values (*p*) for each effect are listed in the right columns. *p<0.05, **p<0.01

| Two-choice Cocaine Self-Administration  Infusions | | **ANOVA**  **df, F-statistic, p-value** | | |
| --- | --- | --- | --- | --- |
| Group | Effect | *df* | *F* | *p* |
| NAc  Unilateral vs. Bilateral | Day | *–* | *–* | *–* |
|  | Day x Unilateral | *–* | *–* | *–* |
|  | Laser | *1, 5* | 8.950 | *0.030** |
|  | Laser x Unilateral | *1, 5* | 2.652 | *0.164* |
|  | Day x Laser | *–* | *–* | *–* |
|  | Day x Laser x Unilateral | *–* | *–* | *–* |
|  | Unilateral | *1, 5* | 1.048 | *0.353* |
| CeA  Unilateral vs. Bilateral | Day | *–* | – | *–* |
|  | Day x Unilateral | *–* | – | *–* |
|  | Laser | *1, 4* | 10.309 | *0.033** |
|  | Laser x Unilateral | *1, 4* | 0.067 | *0.809* |
|  | Day x Laser | *–* | – | *–* |
|  | Day x Laser x Unilateral | *–* | – | *–* |
|  | Unilateral | *1, 4* | 2.024 | *0.228* |
| BNST  Unilateral vs. Bilateral | Day | *–* | – | *–* |
|  | Day x Unilateral | *–* | – | *–* |
|  | Laser | *1, 4* | 0.024 | *0.884* |
|  | Laser x Unilateral | *1, 4* | 0.003 | *0.957* |
|  | Day x Laser | *–* | – | *–* |
|  | Day x Laser x Unilateral | *–* | – | *–* |
|  | Unilateral | *1, 4* | 0.156 | *0.713* |

**Table S9. Unilateral vs. bilateral ChR2 rats in two-choice cocaine self-administration.** The number of earned cocaine infusions for either *Laser-paired* or *Cocaine alone* options in the two-choice self-administration test were compared across the 10 test days in NAc (n=4 unilateral ChR2, n=3 bilateral), CeA (n=2 unilateral ChR2, n=4 bilateral), and BNST (n=3 unilateral ChR2, n=3 bilateral) groups. The first column describes the group being analyzed in the two-way ANOVA. The second column shows the effects tested including the main effect of day (i.e., 1-10 test days), laser (i.e., *Laser+Cocaine* or *Cocaine alone*), effect of unilateral (i.e., ChR2 unilateral or ChR2 bilateral groups), and interactions between these effects. Degrees of freedom (df), F-statistics (*F*), and two-tailed p-values (*p*) for each effect are listed in the right columns. *p<0.05, **p<0.01

| Progressive Ratio  Cocaine Self-Administration | | **ANOVA**  **df, F-statistic, p-value** | | |
| --- | --- | --- | --- | --- |
| Group | Effect | *df* | *F* | *p* |
| NAc  Unilateral vs. Bilateral | Laser | *1, 5* | *6.482* | *0.052* |
|  | Laser x Unilateral | *1, 5* | 2.625 | *0.166* |
|  | Unilateral | *1, 5* | 1.062 | *0.350* |
| CeA  Unilateral vs. Bilateral | Laser | *1, 4* | 53.455 | *0.002*** |
|  | Laser x Unilateral | *1, 4* | 1.091 | *0.355* |
|  | Unilateral | *1, 4* | 0.145 | *0.723* |
| BNST  Unilateral vs. Bilateral | Laser | *1, 4* | 2.897 | *0.187* |
|  | Laser x Unilateral | *1, 4* | 2.074 | *0.245* |
|  | Unilateral | *1, 4* | 29.299 | *0.012** |

**Table S10. Unilateral vs. bilateral ChR2 effects for progressive ratio breakpoints.**

Enhancements in effort breakpoint for the *Laser+Cocaine* progressive ratio test day were evaluated in NAc (n=4 unilateral ChR2, n=3 bilateral), CeA (n=2 unilateral ChR2, n=4 bilateral), and BNST (n=3 unilateral ChR2, n=3 bilateral) groups. The first column describes the group being analyzed in the two-way ANOVA. The second column shows the effects tested including the main effect of laser (i.e., whether the day was for the *Laser+Cocaine* or *Cocaine alone*), effect of unilateral (i.e., ChR2 unilateral or ChR2 bilateral groups), and interactions between these effects. Degrees of freedom (df), F-statistics (*F*), and two-tailed p-values (*p*) for each effect are listed in the right columns. *p<0.05, **p<0.01

| Spout Self-Stimulation | | **ANOVA**  **df, F-statistic, p-value** | | |
| --- | --- | --- | --- | --- |
| Group | Effect | *df* | *F* | *p* |
| NAc  Unilateral vs. Bilateral | Day | *2, 4* | *2.227* | *0.224* |
|  | Day x Unilateral | *2, 4* | 0.314 | *0.747* |
|  | Laser | *1, 5* | 8.769 | *0.031** |
|  | Laser x Unilateral | *1, 5* | 0.057 | *0.821* |
|  | Day x Laser | *2, 4* | 1.128 | *0.409* |
|  | Day x Laser x Unilateral | *2, 4* | 0.034 | *0.967* |
|  | Unilateral | *1, 5* | 0.162 | *0.704* |
| CeA  Unilateral vs. Bilateral | Day | *2, 5* | 0.390 | *0.696* |
|  | Day x Unilateral | *2, 5* | 0.491 | *0.639* |
|  | Laser | *1, 6* | 3.393 | *0.115* |
|  | Laser x Unilateral | *1, 6* | 0.919 | *0.375* |
|  | Day x Laser | *2, 5* | 2.253 | *0.201* |
|  | Day x Laser x Unilateral | *2, 5* | 1.678 | *0.277* |
|  | Unilateral | *1, 6* | 0.312 | *0.597* |
| BNST  Unilateral vs. Bilateral | Day | *2, 5* | 1.072 | *0.41* |
|  | Day x Unilateral | *2, 5* | 1.497 | *0.309* |
|  | Laser | *1, 6* | 0.024 | *0.918* |
|  | Laser x Unilateral | *1, 6* | 0.003 | *0.652* |
|  | Day x Laser | *2, 5* | 0.027 | *0.973* |
|  | Day x Laser x Unilateral | *2, 5* | 1.008 | *0.429* |
|  | Unilateral | *1, 6* | 1.369 | *0.286* |

**Table S11. Unilateral vs. bilateral ChR2 effects for self-stimulation test days 1-3.** The number of interactions with either the *Laser-spout* or the *Inactive-spout* in the spout self-stimulation test were compared across the 3 test days for all NAc (n=4 unilateral ChR2, n=3 bilateral), CeA (n=4 unilateral ChR2, n=4 bilateral), and BNST (n=3 unilateral ChR2, n=5 bilateral) groups. The first column describes the group being analyzed in the two-way ANOVA. The second column shows the effects tested including the main effect of day (i.e., across 3 test days), effect of laser (i.e., whether the response was for the *Laser-spout* or *Inactive-spout*), effect of unilateral (i.e., ChR2 unilateral or ChR2 bilateral groups), and interactions between these effects. Degrees of freedom (df), F-statistics (*F*), and two-tailed p-values (*p*) for each effect are listed in the right columns. *p<0.05, **p<0.01

| Two-choice Cocaine Self-Administration  Infusions | | **ANOVA**  **df, F-statistic, p-value** | | |
| --- | --- | --- | --- | --- |
| Group | Effect | *df* | *F* | *p* |
| NAc  Female vs. Male | Day | *–* | – | – |
|  | Day x Sex | *–* | – | *–* |
|  | Laser | *1, 5* | 10.309 | *0.033** |
|  | Laser x Sex | *1, 5* | 0.067 | *0.809* |
|  | Day x Laser | *–* | – | *–* |
|  | Day x Laser x Sex | *–* | – | – |
|  | Sex | *1, 5* | 2.024 | *0.228* |
| CeA  Female vs. Male | Day | *–* | – | *–* |
|  | Day x Sex | *–* | – | *–* |
|  | Laser | *1, 4* | 9.164 | *0.039** |
|  | Laser x Sex | *1, 4* | 0.233 | *0.655* |
|  | Day x Laser | *–* | – | *–* |
|  | Day x Laser x Sex | *–* | – | *–* |
|  | Sex | *1, 4* | 0.390 | *0.566* |
| BNST  Female vs. Male | Day | *–* | – | *–* |
|  | Day x Sex | *–* | – | *–* |
|  | Laser | *1, 4* | 0.077 | *0.795* |
|  | Laser x Sex | *1, 4* | 0.618 | *0.476* |
|  | Day x Laser | *–* | – | *–* |
|  | Day x Laser x Sex | *–* | – | *–* |
|  | Sex | *1, 4* | 7.607 | *0.051* |

**Table S12. Female vs. male ChR2 rats in two-choice cocaine self-administration.** The number of earned cocaine infusions for either *Laser-paired* or *Cocaine alone* options in the two-choice self-administration test were compared across the 10 test days in NAc (n=5 female ChR2, n=2 male), CeA (n=4 female ChR2, n=2 male), and BNST (n=3 female ChR2, n=3 male) groups. The first column describes the group being analyzed in the two-way ANOVA. The second column shows the effects tested including the main effect of day (i.e., 1-10 test days), laser (i.e., *Laser+Cocaine* or *Cocaine alone*), effect of sex (i.e., ChR2 female or ChR2 male groups), and interactions between these effects. Degrees of freedom (df), F-statistics (*F*), and two-tailed p-values (*p*) for each effect are listed in the right columns. *p<0.05

| Progressive Ratio  Cocaine Self-Administration | | **ANOVA**  **df, F-statistic, p-value** | | |
| --- | --- | --- | --- | --- |
| Group | Effect | *df* | *F* | *p* |
| NAc  Female vs. Male | Laser | *1, 5* | *2.488* | *0.176* |
|  | Laser x Sex | *1, 5* | 0.133 | *0.731* |
|  | Sex | *1, 5* | 0.696 | *0.442* |
| CeA  Female vs. Male | Laser | *1, 4* | 38.264 | *0.003*** |
|  | Laser x Sex | *1, 4* | 0.226 | *0.659* |
|  | Sex | *1, 4* | 0.035 | *0.860* |
| BNST  Female vs. Male | Laser | *1, 3* | 0.682 | *0.469* |
|  | Laser x Sex | *1, 3* | 0.027 | *0.879* |
|  | Sex | *1, 3* | 0.347 | *0.597* |

**Table S13. Female vs. male ChR2 rats progressive ratio breakpoints.**

Enhancements in effort breakpoint for the *Laser+Cocaine* progressive ratio test day were evaluated in (n=5 female ChR2, n=2 male), CeA (n=4 female ChR2, n=2 male), and BNST (n=2 female ChR2, n=3 male) groups. The first column describes the group being analyzed in the two-way ANOVA. The second column shows the effects tested including the main effect of laser (i.e., whether the day was for the *Laser+Cocaine* or *Cocaine alone*), eeffect of sex (i.e., ChR2 female or ChR2 male groups), and interactions between these effects. Degrees of freedom (df), F-statistics (*F*), and two-tailed p-values (*p*) for each effect are listed in the right columns. *p<0.05, **p<0.01

| Spout Self-stimulation | | **ANOVA**  **df, F-statistic, p-value** | | |
| --- | --- | --- | --- | --- |
| Group | Effect | *df* | *F* | *p* |
| NAc  Female vs. Male | Day | *2, 4* | 7.858 | *0.041** |
|  | Day x Sex | *2, 4* | 3.323 | *0.141* |
|  | Laser | *1, 5* | 6.474 | *0.052* |
|  | Laser x Sex | *1, 5* | 0.705 | *0.439* |
|  | Day x Laser | *2, 4* | 1.911 | *0.261* |
|  | Day x Laser x Sex | *2, 4* | 1.228 | *0.384* |
|  | Sex | *1, 5* | 0.440 | *0.537* |
| CeA  Female vs. Male | Day | *2, 5* | 0.212 | *0.816* |
|  | Day x Sex | *2, 5* | 2.246 | *0.201* |
|  | Laser | *1, 6* | 4.169 | *0.087* |
|  | Laser x Sex | *1, 6* | 0.115 | *0.746* |
|  | Day x Laser | *2, 5* | 0.786 | *0.505* |
|  | Day x Laser x Sex | *2, 5* | 3.762 | *0.101* |
|  | Sex | *1, 6* | 1.037 | *0.348* |
| BNST  Female vs. Male | Day | *2, 5* | 1.119 | *0.397* |
|  | Day x Sex | *2, 5* | 2.841 | *0.150* |
|  | Laser | *1, 6* | 0.024 | *0.882* |
|  | Laser x Sex | *1, 6* | 0.439 | *0.532* |
|  | Day x Laser | *2, 5* | 0.079 | *0.925* |
|  | Day x Laser x Sex | *2, 5* | 0.499 | *0.635* |
|  | Sex | *1, 6* | 0.485 | *0.512* |

**Table S14. Female vs. male ChR2 rats in self-stimulation test days 1-3.** The number of interactions with either the *Laser-spout* or the *Inactive-spout* in the spout self-stimulation test were compared across the 3 test days for all NAc (n=5 female ChR2, n=2 male), CeA (n=4 female ChR2, n=3 male), and BNST (n=3 female ChR2, n=5 male) groups. The first column describes the group being analyzed in the two-way ANOVA. The second column shows the effects tested including the main effect of day (i.e., across 3 test days), effect of laser (i.e., whether the response was for the *Laser-spout* or *Inactive-spout*), effect of unilateral (i.e., ChR2 female or ChR2 male groups), and interactions between these effects. Degrees of freedom (df), F-statistics (*F*), and two-tailed p-values (*p*) for each effect are listed in the right columns. *p<0.05

**Lack of correlation in BNST behavioral data.** In BNST ChR2 rats, the degree of laser-paired preference for cocaine in the two-choice task failed to correlate with laser-paired cocaine breakpoint in the progressive ratio task (n=6, *r*=-0.618, *p*=0.191) or the self-stimulation task (n=6, *r*=-0.641, *p*=0.170). Similarly, laser-paired cocaine breakpoints did not correlate with self-stimulation responses (n=6, *r*=-0.344, *p*=0.504).

**Figure S1. Distant Fos effects in male and female ChR2 rats.**


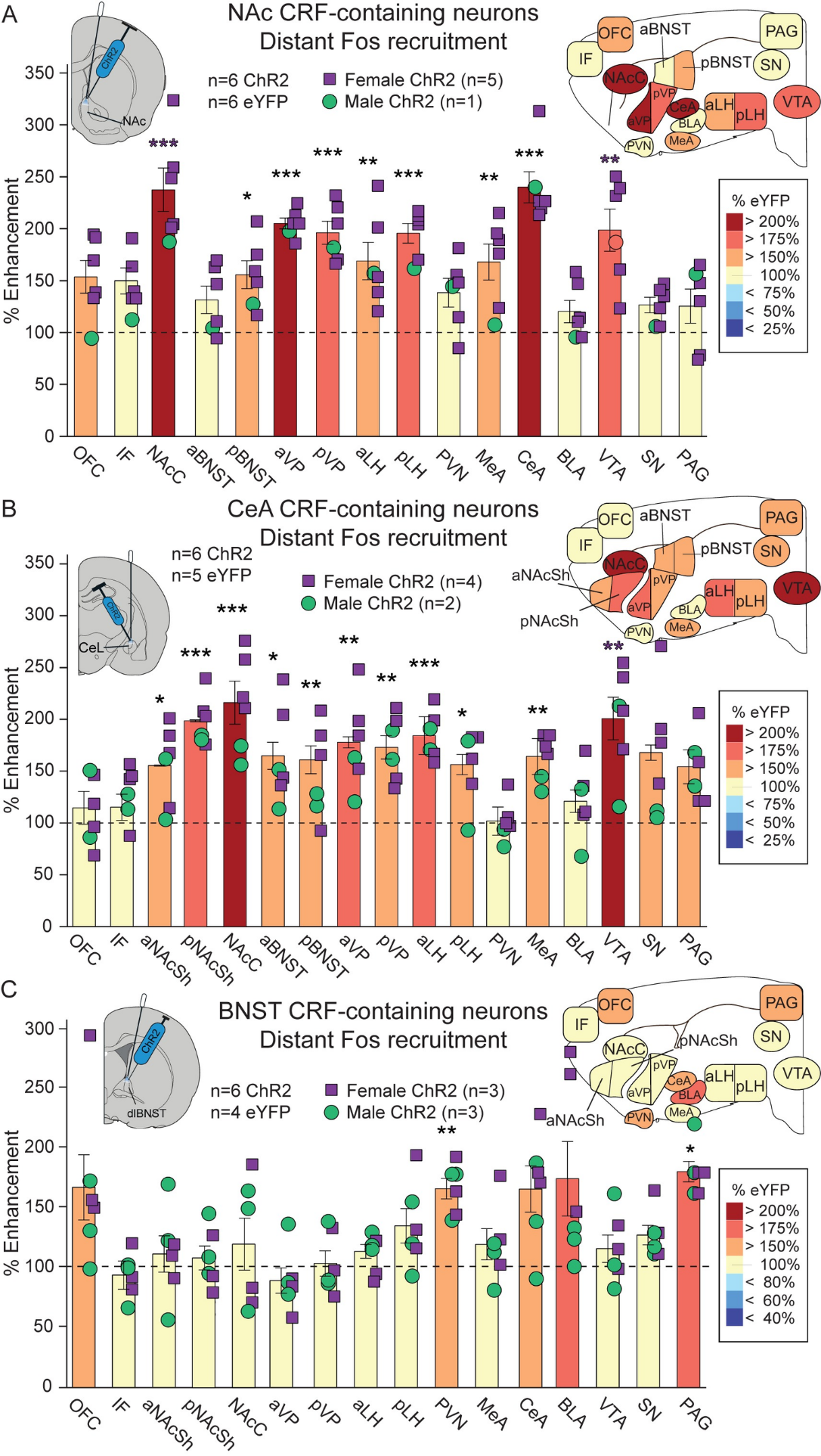


**Figure S2. Two-choice cocaine self-administration in male and female ChR2 rats.**


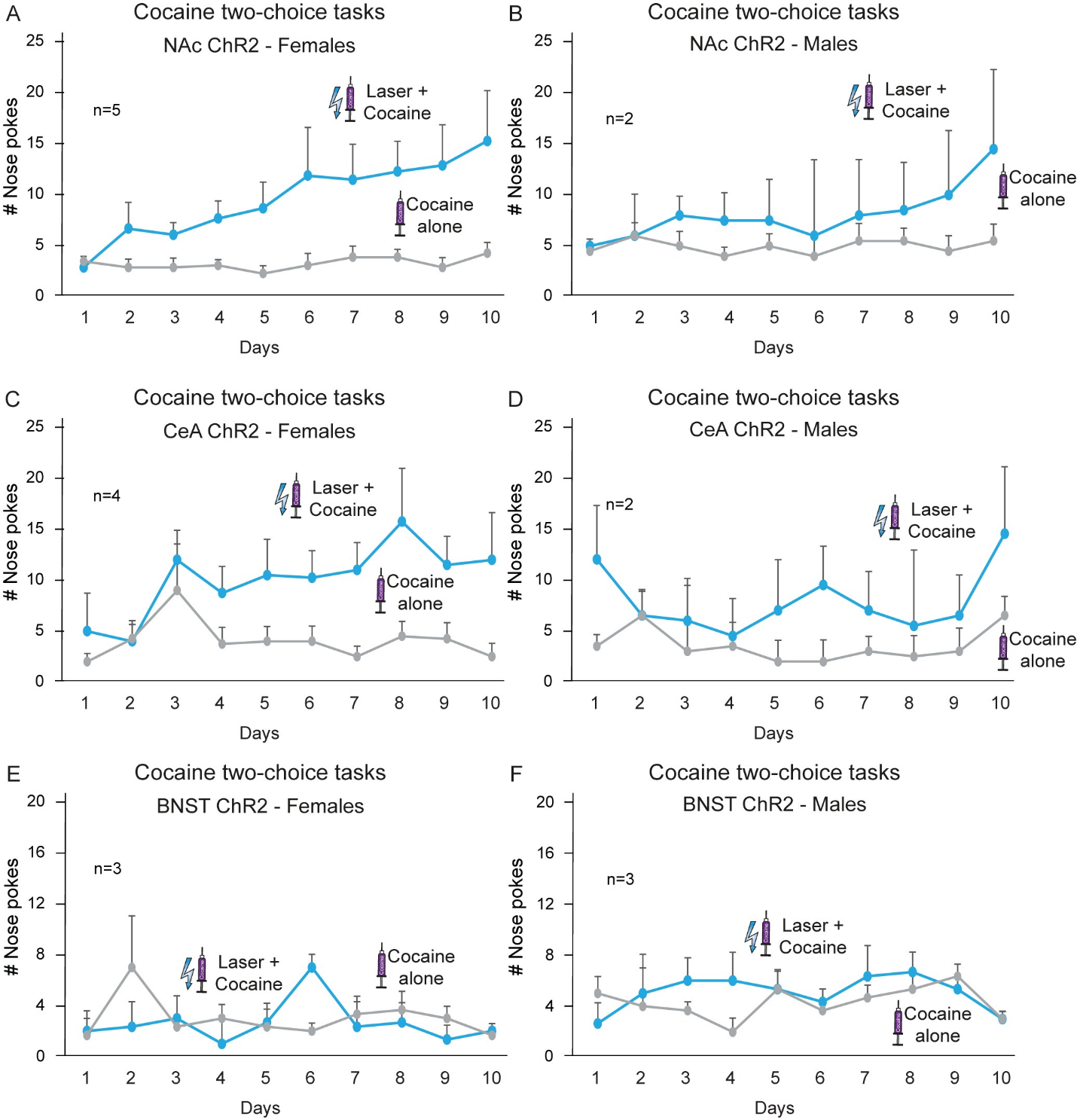


**Figure S3. Progressive ratio breakpoints for cocaine in male and female ChR2 rats.**


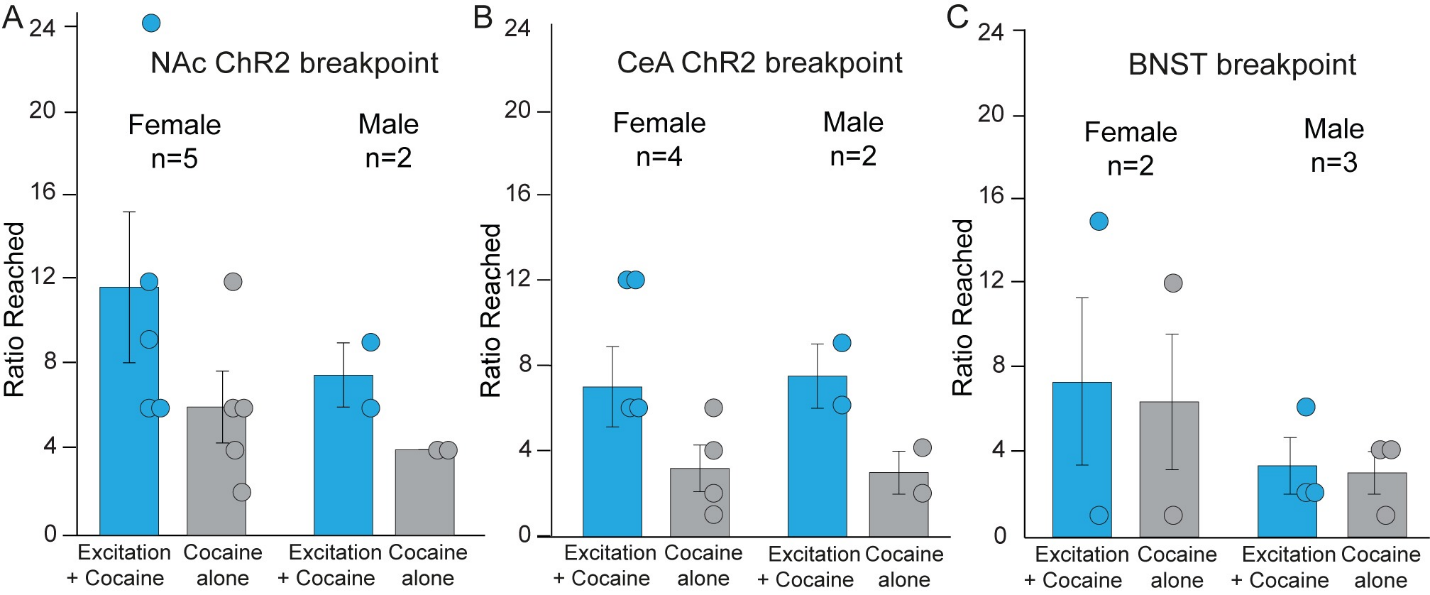


**Figure S4. Spout self-stimulation in male and female ChR2 rats.**


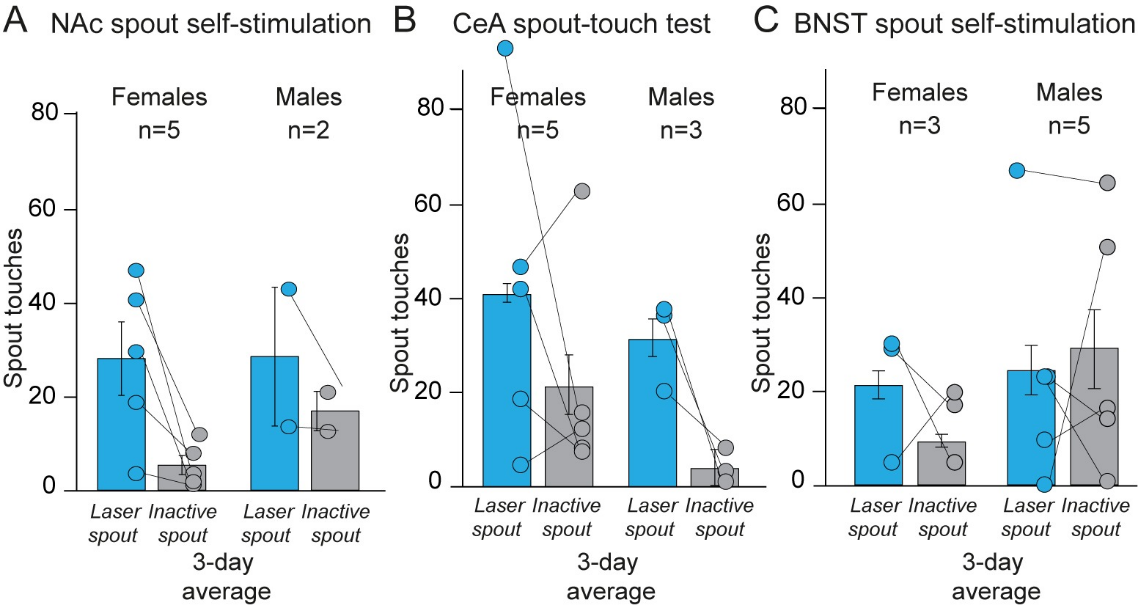


**Figure S5. Example timelines of experimental tests.** Two example timelines that show the order of experimental tests for an individual animal are displayed above. After surgery, rats either started with cocaine self-administration tasks or started with self-stimulation test (counter-balanced order). Cocaine two-choice and progressive ratio tasks always occurred consecutively, either before or after spout self-stimulation testing. The cocaine two-choice task required 10 days of experimental data, though at least one “off” day was provided to rats during the testing period without any experimental testing.


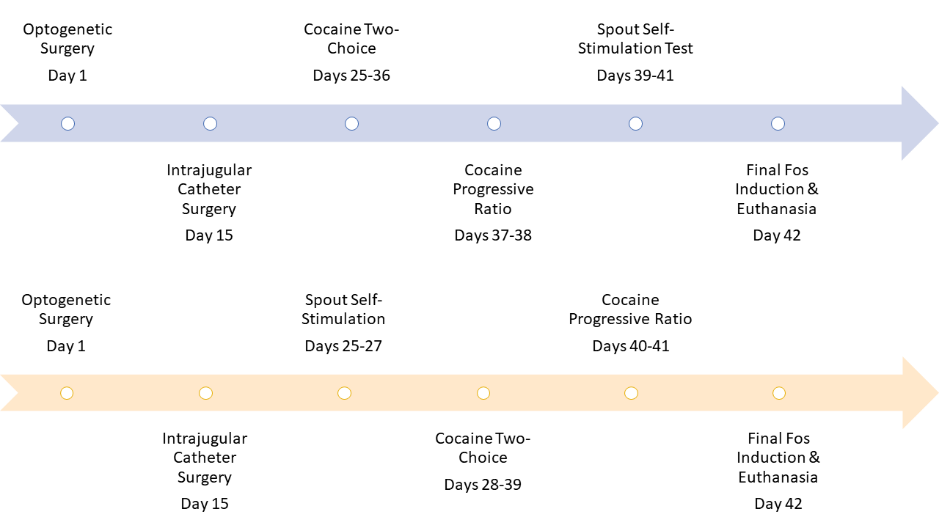

Supplement: S1 File — (DOCX) [file pone.0267345.s001.docx]
